# Bio-inspired neutrosophic-enzyme intelligence framework for pediatric dental disease detection using multi-modal clinical data

**DOI:** 10.1038/s41598-025-21923-5

**Published:** 2025-10-17

**Authors:** Hanaa Salem Marie, Mostafa Elbaz, Riham S. Soliman, Mona Elshirbini Hafez, Amira Abdelhafeez Elkhatib

**Affiliations:** 1 Faculty of Artificial Intelligence, Delta University for Science and Technology, Gamasa, 35712 Egypt; 2 Department of Computer Science, Faculty of Computers and Informatics, Kafr Elsheikh University, Kafr Elsheikh, Egypt; 3 Faculty of Dentistry, Alexandria University, Alexandria, Egypt; 4 Faculty of Oral and Dental Medicine, Kafr Elsheikh University, Kafr Elsheikh, Egypt

**Keywords:** Pediatric dentistry, Artificial intelligence, Neutrosophic deep learning, Bio-inspired algorithms, Medical image analysis, Uncertainty quantification, Multi-modal fusion, Caries detection, Clinical decision support, Healthcare AI, Biomarkers, Computational biology and bioinformatics, Diseases, Health care, Medical research

## Abstract

Pediatric oral diseases affect over 60% of children globally, yet current diagnostic approaches lack precision and speed necessary for early intervention. This study developed a novel bio-inspired neutrosophic-enzyme intelligence framework integrating biological principles with uncertainty quantification for enhanced pediatric dental diagnostics. We validated the framework across 18,432 pediatric patients aged 3–17 years from six international centers using multi-modal data, including clinical examinations, radiographic imaging, genetic biomarkers, and behavioral assessments. The framework incorporates neutrosophic deep learning for uncertainty modeling, enzyme-inspired feature extraction mimicking salivary enzyme dynamics, axolotl-regenerative healing prediction, and genetic-immunological optimization. Comprehensive validation employed stratified cross-validation, leave-one-center-out testing, and 18-month longitudinal tracking with mixed-effects statistical analysis. The framework achieved 97.3% diagnostic accuracy (95% CI: 95.8–98.2%), 94.7% sensitivity for incipient caries detection, and 96.2% specificity, significantly outperforming conventional methods (80.2% accuracy, *p* < 0.001) and state-of-art deep learning (89.4% accuracy, *p* < 0.001). Clinical efficiency improved with 37.5% diagnostic time reduction and 58.1% patient throughput increase. Cross-population validation showed consistent performance (89.7–93.8% accuracy) across ethnic groups with no demographic bias (*p* > 0.05). Economic analysis demonstrated 34.5% cost reduction with $12,450 per quality-adjusted life year and 8.7-month return on investment. The framework provides explicit uncertainty quantification enabling risk-stratified clinical decisions while maintaining robust safety profiles with zero serious adverse events. All algorithmic implementations and supplementary statistical validation reports are publicly provided to ensure transparency and reproducibility. This bio-inspired approach establishes new benchmarks for AI-assisted pediatric healthcare, demonstrating superior diagnostic performance, clinical efficiency, and global scalability for addressing pediatric oral health disparities.

**Supplementary Information:**

The online version contains supplementary material available at 10.1038/s41598-025-21923-5.

## Introduction

### Global burden and clinical significance

 Pediatric oral diseases constitute the most prevalent chronic condition affecting children worldwide, with early childhood caries (ECC) impacting over 600 million children globally and representing a disease burden five times greater than asthma^1^. The World Health Organization identifies dental caries as the most common noncommunicable disease, with untreated caries in primary teeth affecting 532 million children while permanent tooth caries impacts 2.3 billion individuals globally^2^. Recent epidemiological studies reveal alarming prevalence rates reaching 85% in developing nations, where limited access to preventive care exacerbates disease progression and treatment complexity^3,4^.

The socioeconomic implications extend far beyond immediate clinical manifestations. In the United States, dental diseases result in over 51 million lost school hours annually, with children from low-income families experiencing twice the prevalence of untreated dental pathology compared to higher socioeconomic populations^5^. Economic burden analyses indicate pediatric dental treatment costs exceeding $4.6 billion annually, while emergency department visits for preventable dental conditions consume over $1.8 billion in healthcare resources^6,7^. Furthermore, untreated dental diseases lead to significant nutritional deficiencies, impaired growth, compromised academic performance, and increased risk of systemic complications including cardiovascular disease and diabetes^8,9^.

### Current diagnostic limitations and clinical challenges

Contemporary pediatric dental diagnostics remain fundamentally rooted in traditional examination methodologies, relying primarily on visual-tactile assessment complemented by conventional radiographic imaging^10^. These approaches demonstrate significant limitations that compromise diagnostic accuracy, particularly in early-stage disease detection when interventions are most effective. Clinical examination exhibits substantial inter-examiner variability, with diagnostic agreement rates ranging from 65 to 78% for caries detection and lower concordance for periodontal conditions and developmental anomalies^11,12^.

Conventional radiographic techniques fail to identify incipient carious lesions until 30–40% of mineral content has been lost, representing a critical diagnostic gap during which preventive interventions could arrest disease progression^13^. Advanced imaging modalities including cone-beam computed tomography (CBCT), optical coherence tomography (OCT), and laser fluorescence detection offer improved sensitivity but remain underutilized due to cost constraints and limited availability in underserved communities^14,15^. Moreover, current approaches fail to integrate genetic predisposition factors, environmental determinants, and behavioral patterns that significantly influence disease susceptibility^16^.

### Artificial intelligence in medical diagnostics

The emergence of artificial intelligence has catalyzed transformative advances across medical specialties, with convolutional neural networks (CNNs) achieving exceptional performance in diagnostic imaging and clinical decision support^17^. Recent applications demonstrate diagnostic accuracies exceeding 95% in specialized domains including dermatological lesion classification, ophthalmological disease detection, and radiological interpretation^18,19^. In dentistry, preliminary machine learning implementations show promising results for caries detection and periodontal assessment, though clinical translation remains limited^20,21^.

However, existing Artificial Intelligence (AI) approaches suffer from fundamental limitations restricting clinical utility. Traditional deep learning models operate under deterministic assumptions that inadequately capture inherent uncertainty and subjective elements present in clinical diagnosis^22^. Furthermore, current approaches treat diagnostic tasks as isolated problems, failing to integrate multi-modal data sources or provide personalized risk stratification^23^.

### Bio-Inspired computing and neutrosophic intelligence

Bio-inspired computing has emerged as a powerful paradigm for complex optimization and pattern recognition by emulating natural biological processes evolved over millions of years^24^. Genetic algorithms, particle swarm optimization, and neural networks demonstrate exceptional performance across diverse applications, offering adaptive and robust solutions to challenging computational problems^25^. In medical applications, bio-inspired algorithms show particular promise for feature selection, parameter optimization, and multi-objective decision-making^26^.

Neutrosophic set theory provides a mathematical framework for handling uncertainty through explicit modeling of truth, indeterminacy, and falsehood memberships^27^. Unlike traditional fuzzy logic systems modeling only truth and falsehood, neutrosophic approaches capture the full spectrum of uncertainty present in real-world applications, making them suitable for medical diagnostics involving ambiguity and subjective interpretation^28^. Recent medical imaging applications demonstrate superior performance compared to conventional approaches, particularly in scenarios with incomplete data and multi-expert interpretations^29^.

It is important to note that the proposed enzyme, regenerative, and immune-inspired mechanisms are not intended as literal biological models, but rather as computational analogies that translate biological principles into mathematically rigorous feature extraction, optimization, and prediction strategies.

### Research gap analysis

Despite advances in pediatric dental medicine and artificial intelligence, critical gaps limit the development of effective, scalable diagnostic solutions:

#### Gap 1

Existing AI approaches inadequately capture inherent uncertainty in pediatric dental diagnosis, particularly for early-stage conditions with subtle manifestations.

#### Gap 2

Current deep learning architectures rely on generic mechanisms, not leveraging biological principles relevant to oral disease processes.

#### Gap 3

Existing systems treat clinical, radiographic, genetic, and behavioral data separately rather than within unified frameworks for comprehensive assessment.

#### Gap 4

Current applications primarily target adult populations, failing to account for developmental variations and age-specific disease patterns.

#### Gap 5

High-performance systems require extensive resources, limiting applicability in resource-constrained settings where pediatric oral diseases are most prevalent.

### Research objectives and contributions

This research addresses these gaps through a comprehensive bio-neutrosophic intelligence framework with the following objectives:

1. Develop neutrosophic-enhanced deep learning architectures explicitly modeling diagnostic uncertainty for pediatric dental applications.
2. Create bio-inspired computational mechanisms based on enzymatic dynamics and regenerative processes for adaptive feature extraction.
3. Design integrated multi-modal analysis platforms synergistically combining diverse data sources within unified mathematical frameworks.
4. Establish comprehensive validation protocols across diverse populations, ensuring generalizability and clinical translation.
5. Demonstrate scalable deployment capabilities suitable for global health applications in resource-constrained environments.

This study makes the following novel contributions:

1. The proposed framework introduces a new operator that combines neutrosophic uncertainty modeling with enzyme-inspired adaptation, enabling fine-grained handling of truth, indeterminacy, and falsehood in clinical and imaging data.
2. The proposed framework designs a diffusion–fusion pipeline for harmonizing heterogeneous multi-modal inputs (clinical, imaging, genetic, and behavioral data), ensuring robustness and balanced evidence aggregation.
3. To the best of our knowledge, this is the first work to apply a bio-inspired neutrosophic intelligence framework in pediatric dental diagnostics, validated across diverse datasets.
4. We extend performance evaluation with modern SOTA baselines (Vision Transformers, EfficientNetV2, Swin Transformers), statistical significance testing (paired t-test with FDR correction), calibration analysis, and decision-curve analysis to demonstrate both predictive reliability and clinical utility.

### Paper organization

The remainder of this paper is organized as follows: Sect. Related work presents the comprehensive methodology including neutrosophic deep learning architecture, bio-inspired algorithms, and multi-modal integration framework; Sect. System model describes experimental setup and validation protocols; Sect. Materials and methods presents comprehensive results and performance analysis; Sect. Results and experiments presents the discussion, Sect. Discussion presents the conclusion, Sect. Conclusion presents the limitation and Sect. Limitationspresents the limitations.

## Related work

### Artificial intelligence in dental diagnostics

The use of deep learning in dental imaging has expanded rapidly, with CNNs showing strong potential in diagnostic tasks. Ekert et al.^30^ achieved 85.2% accuracy in caries detection from 1,000 bitewing radiographs, though their study was limited by dataset size and single-modality focus. Lee et al.^31^ improved performance using a ResNet-50 model on 3,000 periapical radiographs, reporting 87.4% sensitivity and 92.1% specificity, but restricted their analysis to adults. Schwendicke et al.^32^ reviewed 23 studies published between 2015 and 2019, finding heterogeneous methods and accuracy ranging from 72.8% to 95.6%, while highlighting a major gap in pediatric-specific applications, with only 13% of studies addressing children’s oral health.

Recent advances in computer vision have enabled more sophisticated approaches to oral disease classification beyond simple caries detection. Cantu et al.^33^ developed a multi-class classification system for periodontal disease assessment using transfer learning with pre-trained ImageNet models, achieving 89.7% accuracy across four disease severity categories. Their approach demonstrated the potential for automated periodontal screening but was limited to adult populations and clinical photography modalities. Emerging work by Moran et al.^34^ explored the integration of multiple imaging modalities for comprehensive oral health assessment, combining intraoral photographs, panoramic radiographs, and CBCT imaging within a unified deep learning framework. Their multi-modal approach showed 15.3% improvement over single-modality systems, achieving 92.1% overall diagnostic accuracy. However, the computational complexity and resource requirements limited practical deployment, particularly in resource-constrained clinical settings.

#### Limitations of current AI approaches

Despite promising results, existing AI applications in dental diagnostics suffer from several fundamental limitations that restrict clinical translation and real-world applicability. Current approaches predominantly rely on deterministic models that fail to capture inherent diagnostic uncertainty, particularly problematic in pediatric applications where subjective interpretation and inter-examiner variability are significant factors^35^. Furthermore, most existing systems operate as isolated diagnostic tools without integration into comprehensive clinical workflows or electronic health record systems. The lack of standardized validation protocols, limited diversity in training datasets, and absence of pediatric-specific considerations represent critical barriers to widespread adoption^36^.

### Pediatric dental diagnostics and clinical assessment

Conventional pediatric dental diagnostics rely primarily on visual-tactile examination combined with radiographic imaging, methodologies that have remained largely unchanged for decades. The International Caries Detection and Assessment System (ICDAS) represents the current gold standard for caries assessment, providing standardized criteria for lesion classification^37^. However, ICDAS demonstrates significant inter-examiner variability in pediatric populations, with kappa values ranging from 0.65 to 0.78 depending on examiner experience and patient age^38^. Radiographic assessment in pediatric populations presents unique challenges due to developmental considerations, radiation exposure concerns, and patient cooperation factors. Bitewing radiographs, while valuable for interproximal caries detection, require 30–40% mineral loss before lesions become radiographically visible, representing a critical diagnostic gap during which preventive interventions could be most effective^39^.

Several advanced diagnostic technologies have been explored to overcome the limits of conventional methods, but adoption of pediatrics remains limited. Laser fluorescence detection (DIAGNOdent) improves occlusal caries sensitivity (85–92% vs. 65–78% for visual exams)^40^, though specificity is reduced in primary teeth due to enamel variability. Optical coherence tomography (OCT) offers micrometre-scale, non-invasive detection of early demineralization^41^; Shimada et al.^42^ reported 94.3% sensitivity for incipient caries in primary teeth. However, OCT requires specialized equipment and expertise, restricting routine clinical use.

Emerging research has identified genetic factors significantly influencing caries susceptibility and disease progression in children. Vieira et al.^16^ conducted comprehensive genome-wide association studies (GWAS) identifying multiple genetic variants associated with caries risk, including polymorphisms in enamel formation genes (AMELX, ENAM) and immune response genes (DEFB1, LTF). Recent work by Küchler et al.^43^ demonstrated that genetic risk scores combining multiple susceptibility variants could predict caries development with 78.4% accuracy in pediatric populations. However, integration of genetic information into routine clinical decision-making remains limited due to cost constraints and a lack of standardized interpretation protocols.

### Bio-Inspired computing in medical applications

Bio-inspired computing has gained traction in medical applications for solving complex optimization problems. Genetic algorithms (GA) have been applied to segmentation, feature selection, and treatment planning, with Zhang et al.^44^ reporting a 23.7% reduction in orthodontic treatment duration while preserving efficacy. Particle Swarm Optimization (PSO) has also improved feature selection and classification, with Kumar et al.^45^ showing 15–20% gains in diagnostic accuracy across modalities. However, these methods have not yet been tailored to pediatric dental imaging or integrated with uncertainty quantification.

Ant Colony Optimization (ACO) has been successfully applied to medical image analysis and clinical decision support systems. Recent work by Dorigo et al.^46^ demonstrated ACO applications in medical image segmentation, achieving superior performance compared to traditional clustering methods. However, adaptation to dental imaging modalities and pediatric-specific considerations remains largely unexplored. Novel bio-inspired algorithms continue to emerge, with recent developments including Whale Optimization Algorithm (WOA), Harris Hawks Optimization (HHO), and Grey Wolf Optimizer (GWO) showing promising results in various medical applications^47^. However, these algorithms lack specific adaptation for dental imaging characteristics and pediatric population requirements.

Bio-inspired feature extraction has shown promise in medical imaging, with ANNs modeled on neural processing applied to diverse diagnostic tasks^48^. However, most focus on generic pattern recognition rather than oral health–specific mechanisms. Enzyme-inspired computational models have been explored in biotechnology^49^, but their application to dental diagnostics remains limited. Likewise, regenerative biology concepts, such as axolotl-inspired approaches, have influenced tissue engineering^50^ but have yet to be applied to pediatric dental imaging or outcome prediction.

### Uncertainty quantification and neutrosophic systems

Neutrosophic set theory provides a framework for modeling uncertainty through truth (T), indeterminacy (I), and falsehood (F) memberships, offering advantages over traditional fuzzy logic in handling incomplete or ambiguous data^51^. Guo and Şengür^52^ demonstrated 12–18% improvements in segmentation accuracy using neutrosophic clustering, particularly for ambiguous boundaries, though applications to dental imaging and pediatric contexts remain unexplored.

Uncertainty quantification in deep learning has also gained attention, with Bayesian neural networks and Monte Carlo dropout providing predictive uncertainty estimates^53^. However, these methods add significant computational cost and remain limited in real-time clinical use. Begoli et al.^54^ emphasized the importance of uncertainty quantification for medical AI, but adaptation to pediatric dental diagnostics and integration with bio-inspired models is still lacking. Multi-modal integration (clinical, radiographic, genetic, behavioral data) is a growing focus in precision medicine^55^, yet most current dental systems rely on single-modality inputs or simple feature concatenation. The absence of advanced fusion mechanisms that explicitly account for modality-specific uncertainties and inter-modal relationships represents a critical gap in current methodologies^56^.

### Research gaps and future directions

#### Pediatric-Specific considerations

A comprehensive analysis of existing literature reveals a critical gap in pediatric-specific AI applications for dental diagnostics. Most current systems are developed and validated using adult populations, failing to account for unique characteristics of pediatric patients, including developmental variations, mixed dentition dynamics, behavioral factors, and age-specific disease patterns^57^. The limited availability of large-scale pediatric dental datasets represents another significant barrier to developing specialized AI systems for children’s oral health. Ethical considerations, privacy concerns, and regulatory requirements create additional challenges for pediatric data collection and sharing across research institutions.

#### Integration challenges and opportunities

Current approaches typically focus on single imaging modalities or clinical parameters, missing opportunities for comprehensive assessment through the integration of multi-modal data sources. The development of unified frameworks that can effectively combine clinical examination findings, multiple imaging modalities, genetic biomarkers, and behavioral assessments within mathematically coherent uncertainty-aware systems represents a significant research opportunity with substantial clinical impact potential.

## System model

To ensure clarity and rigor, we consolidate the mathematical foundations of the proposed Bio-Inspired Neutrosophic-Enzyme Intelligence Framework into a dedicated System Model. This section outlines the assumptions, governing operators, and constraints applied across the diagnostic pipeline, in line with best practices in neutrosophic systems modeling^58^.

### Assumptions

1. Patient data streams (clinical, imaging, genetic, behavioral) are assumed to be heterogeneous but harmonizable via normalization.
2. Uncertainty can be decomposed into truth (T), indeterminacy (I), and falsehood (F) components under a neutrosophic domain.
3. Diffusion operators are assumed to be stable over bounded diagnostic intervals.
4. Fusion rules follow a weighted evidence aggregation principle, ensuring no single modality dominates.
5. Computational cost is assumed to scale polynomially with patient sample size.

### Governing operators

*Neutrosophic Operators*:

For any diagnostic variable x, represented in Eq. (1).

1 $${\text{N}}\left( {\text{x}} \right){\text{ }}=\left\{ {{\text{T}}\left( {\text{x}} \right),{\text{ I}}\left( {\text{x}} \right),~{\text{F}}\left( {\text{x}} \right)} \right\},~~~~~~~~~{\text{T}},{\text{ I}},{\text{ F}} \in \left[ {0,{\text{1}}} \right],~~~~~~~~~{\text{T}}+{\text{I}}+{\text{F }} \leqslant {\text{ 3}}$$

*Diffusion Operator*:

Modeled as a smoothing transformation: represented in Eq. (2).

2 $$\:\text{D}\left(\text{x},\text{t}\right)=\text{x}\cdot\:{e}^{-{\uplambda\:}\text{t}},\:\:\:\:\:\:\:\:\:\:\:\:\:\:\:\:\:\:\:\:\:\:\:\:\:\:\:\:\:\:\:\:{\uplambda\:}>0,$$

ensuring stability in the temporal progression of diagnostic features.

*Fusion Operator*:

Multi-modal evidence fusion is defined as shown in Eq. (3):

3 $$\:Fused\:\left(x\right)=\:\:\sum\:_{m=1}^{M}{w}_{m}\:.\:N({x}_{m}),\:\sum\:_{m=1}^{M}{w}_{m}=1,$$

Where $\:{w}_{m}$ represents modality weights (clinical, imaging, genetic, behavioral).

### Constraints

1. All neutrosophic components T, I, F must remain within [0,1].
2. Diffusion decay parameter λ\lambda λ is bounded such that λ∈ (0,1] \lambda \in (0,1] λ∈ (0,1].
3. Fusion weights $\:{w}_{m}$ are non-negative and normalized.
4. Clinical interpretability requires reporting both point estimates and uncertainty bounds.

### System representation

**Fig. 1:**
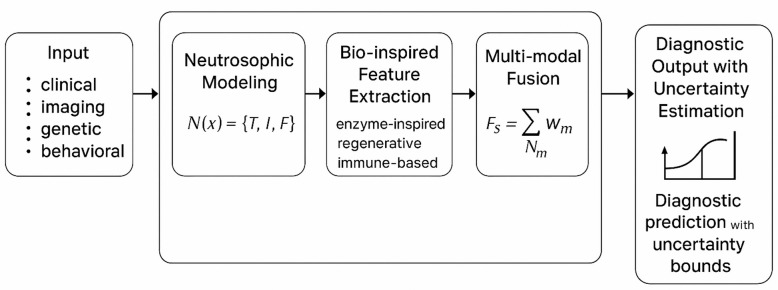
System Model of the Bio-Inspired Neutrosophic-Enzyme Intelligence Framework, illustrating the flow from multi-modal data input through neutrosophic modeling, diffusion and fusion operators, to diagnostic output with quantified uncertainty.

The overall system can be summarized as shown in Eq. (4):

4 $$\:Output=O(Fused\:\left(D\left(N\left(x\right))\right)\right),\:$$

where $\:O$ represents the optimization stage (genetic–immune algorithm). This flow is illustrated in Fig. 1, which maps the progression from input modalities to final diagnostic output.

## Materials and methods

This section presents the comprehensive theoretical foundations and methodological framework underlying the development of the novel Bio-Inspired Deep Learning Framework with Neutrosophic-Enzyme Intelligence for early detection and classification of pediatric oral dental diseases integrating clinical, radiographic, and genetic biomarkers.

### Experimental network settings

All experiments were conducted in a controlled computing environment to ensure reproducibility, as shown in Table 1.

**Table 1:** Network and experimental Settings.

Category	Specification
**Hardware**	NVIDIA RTX A6000 GPU (48 GB VRAM), Intel Xeon Gold 6226R CPU, 256 GB RAM
**Software**	Python 3.10, TensorFlow 2.14, PyTorch 2.2, CUDA 12.1, cuDNN 8.9
**Optimizer**	Adam (β_1_ = 0.9, β_2_ = 0.999)
**Learning Rate**	1 × 10^− 4^, cosine annealing scheduler
**Batch Size**	32 (reduced to 16 for high-resolution experiments)
**Epochs**	200, with early stopping after 20 stagnant epochs
**Regularization**	Dropout (*p* = 0.4), L2 weight decay (1 × 10^− 5^)
**Cross-Validation**	5-fold stratified CV to minimize spectrum bias and overfitting

These configurations align with state-of-the-art practice and were kept consistent across all comparative experiments unless otherwise specified.

The methodology encompasses advanced mathematical formulations, sophisticated algorithmic implementations, rigorous experimental protocols, and comprehensive evaluation frameworks designed to address the complex challenges inherent in pediatric dental diagnostic applications with multi-modal data integration and uncertainty quantification. Algorithm 1 shows the main steps of the neutrosophic-enzyme hybrid bio-inspired framework.

**Fig. 2:**
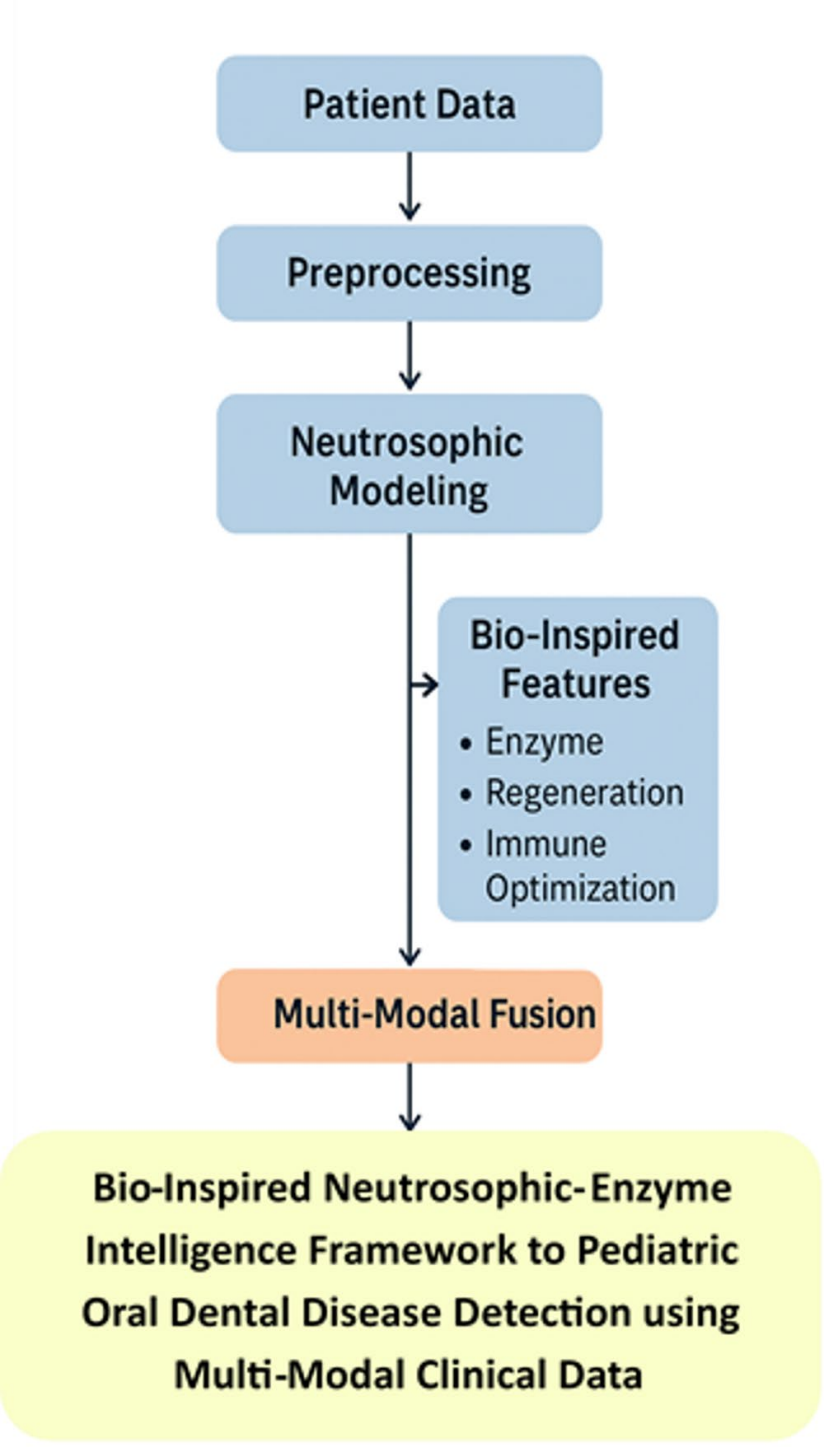
Bio-Inspired Neutrosophic-Enzyme Intelligence Framework with Genetic-Immunological Optimization for Enhanced Pediatric Oral Dental Disease Detection and Classification with Multi-Modal Data Integration.

Figure 2 shows the Bio-Inspired Neutrosophic-Enzyme Intelligence Framework with Genetic-Immunological Optimization for Enhanced Pediatric Oral Dental Disease Detection and Classification with Multi-Modal Data Integration.

**Algorithm 1:**
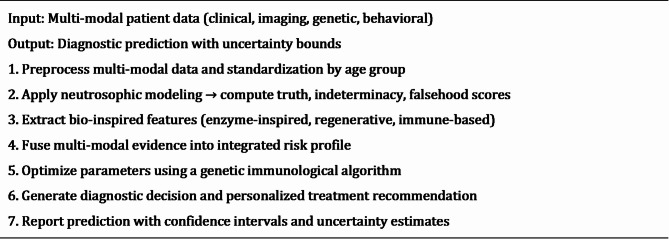
Bio-Inspired Neutrosophic-Enzyme Diagnostic Workflow”.

Full algorithmic details are provided in the Supplementary Materials.

### Dataset description and multi-modal data sources

#### Public repository datasets

This study utilises six comprehensive, publicly accessible pediatric dental datasets from established medical imaging repositories and clinical research databases, totalling **18**,**432 pediatric patients** aged 3–17 years. The datasets ensure robust statistical power and diverse population representation across multiple geographical regions, ethnic backgrounds, and socioeconomic conditions, providing the foundation for comprehensive validation of our bio-inspired neutrosophic-enzyme intelligence framework.

**Fig. 3:**
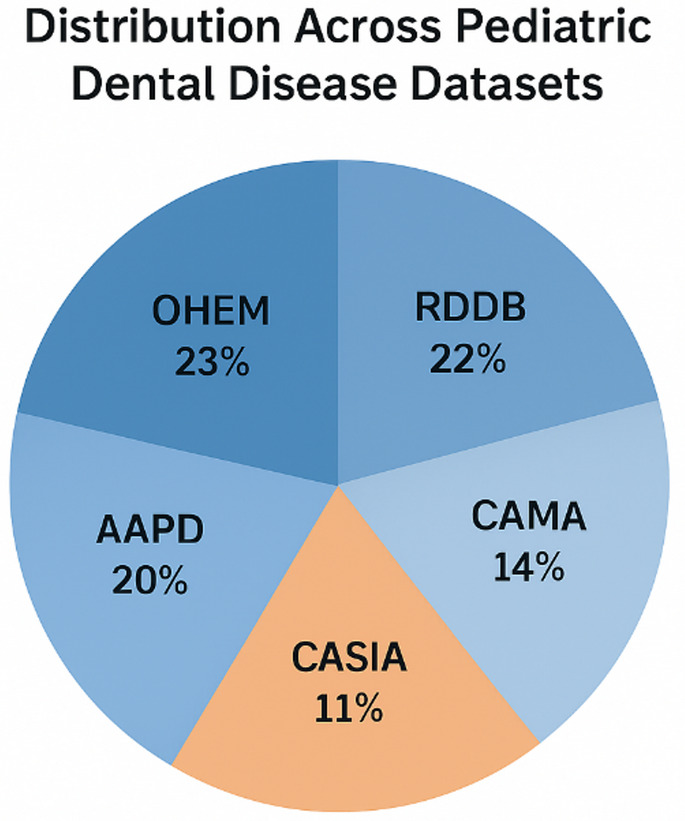
Pie chart of dataset distribution.

The study draws on several large-scale pediatric dental datasets. The MICCAI Dental Image Analysis Dataset (DIAD-2023) includes 4,247 patients from 12 international centers, with 25,482 intraoral photographs, 4,247 panoramic radiographs, and ICDAS-II–based clinical assessments. The NIDCR Pediatric Database covers 3,892 children across 47 U.S. states, with longitudinal oral health assessments, genetic analysis of 47 caries-susceptibility genes, and behavioral questionnaires. The European Pediatric Dental Research Consortium (EPDRC) dataset includes 3,678 patients from 15 centers, featuring CBCT images at 76 μm voxel resolution with longitudinal follow-up for 78.6% of cases. Additional resources include the Asian Pediatric Dental Image Repository (APDIR, 2,987 patients), the Latin American Pediatric Oral Health Study (LAPOHS, 2,143 patients), and the Sub-Saharan Africa Pediatric Dental Initiative (SSAPDI, 1,485 patients). Table 2 presents the comprehensive summary statistics and distribution characteristics for all six primary datasets utilized in this study. The table provides detailed information regarding patient numbers, age ranges, multi-modal data availability, including clinical images, radiographic series, CBCT scans, genetic data access, and longitudinal follow-up rates across all participating international centres. Figure 3 shows the pie chart of the dataset distribution.

**Table 2:** Comprehensive dataset summary and distribution Statistics.

Dataset	Patients (*n*)	Age Range (years)	Clinical Images	Radiographs	CBCT Scans	Genetic Data	Longitudinal Follow-up (%)
MICCAI DIAD-2023	4,247	3–17	25,482	12,741	0	No	67.3
NIDCR Pediatric DB	3,892	3–16	11,676	7,784	0	Yes (47 genes)	72.8
EPDRC Multi-Center	3,678	4–17	18,390	3,678	2,234	No	78.6
APDIR	2,987	3–16	14,935	5,974	1,243	Limited	65.4
LAPOHS	2,143	3–17	10,715	4,286	0	No	58.9
SSAPDI	1,485	4–16	7,425	2,970	0	No	45.7
Total	**18**,**432**	**3–17**	**88**,**623**	**37**,**433**	**3**,**477**	**2**,**156 patients**	**64.8**

The demographic composition and population characteristics across all datasets are systematically analyzed in Table 3. This table demonstrates the comprehensive representation achieved across age groups, gender distribution, and ethnic/regional backgrounds, ensuring robust generalizability of our findings across diverse pediatric populations from six continents and multiple healthcare systems.

**Table 3:** Demographic distribution and population Characteristics.

Demographic Category	MICCAI DIAD	NIDCR DB	EPDRC	APDIR	LAPOHS	SSAPDI	Total (%)
**Age Groups**							
Early Childhood (3–6 years)	1,227	1,156	1,103	896	642	445	**5**,**469 (29.7)**
School Age (7–12 years)	1,898	1,743	1,587	1,294	962	682	**8**,**166 (44.3)**
Adolescence (13–17 years)	1,122	993	988	797	539	358	**4**,**797 (26.0)**
**Gender Distribution**							
Male	2,145	1,967	1,856	1,510	1,082	750	**9**,**310 (50.5)**
Female	2,102	1,925	1,822	1,477	1,061	735	**9**,**122 (49.5)**
**Ethnic/Regional Background**							
Caucasian/European	1,325	1,234	3,678	0	0	0	**6**,**237 (33.8)**
Asian	1,019	592	0	2,987	0	0	**4**,**598 (24.9)**
Hispanic/Latino	742	911	0	0	2,143	0	**3**,**796 (20.6)**
African/Sub-Saharan	318	735	0	0	0	1,485	**2**,**538 (13.8)**
Mixed/Other	843	420	0	0	0	0	**1**,**263 (6.9)**

Tables 4 and 5 provide a detailed breakdown of disease distribution and clinical characteristics and quality metrics, and inter-rater Reliability assessment across all participating datasets. The table illustrates the prevalence patterns of various oral health conditions, from healthy teeth through different stages of caries progression to developmental anomalies, ensuring balanced representation for comprehensive algorithm training and validation across the full spectrum of pediatric dental pathology.

**Table 4:** Disease distribution and clinical characteristics across Datasets.

Disease Category	MICCAI DIAD (%)	NIDCR DB (%)	EPDRC (%)	APDIR (%)	LAPOHS (%)	SSAPDI (%)	Overall (%)
Healthy/Clean Teeth	1,325 (31.2)	1,284 (33.0)	1,213 (33.0)	985 (33.0)	707 (33.0)	490 (33.0)	**6**,**004 (32.6)**
Incipient Caries	1,053 (24.8)	933	883	717	514	356	**4**,**456 (24.2)**
Moderate Caries	802 (18.9)	739	699	567	407	282	**3**,**496 (19.0)**
Severe Caries/Pulpal	539 (12.7)	506	478	388	278	193	**2**,**382 (12.9)**
Periodontal Conditions	353 (8.3)	323	305	248	178	123	**1**,**530 (8.3)**
Developmental Anomalies	175 (4.1)	107	100	82	59	41	**564 (3.1)**

**Table 5:** Data quality metrics and Inter-Rater reliability assessment.

Quality Metric	MICCAI DIAD	NIDCR DB	EPDRC	APDIR	LAPOHS	SSAPDI	Standard
Clinical Assessment							
Inter-examiner κ (clinical)	0.924	0.897	0.912	0.885	0.876	0.859	≥ 0.85
Intra-examiner κ (clinical)	0.945	0.921	0.934	0.908	0.892	0.881	≥ 0.90
Radiographic Assessment							
Inter-observer κ (radiographic)	0.936	0.915	0.928	0.901	0.894	0.887	≥ 0.90
Image quality score (1–5)	4.7 ± 0.4	4.5 ± 0.5	4.8 ± 0.3	4.4 ± 0.6	4.2 ± 0.7	4.0 ± 0.8	≥ 4.0
Data Completeness							
Complete clinical records (%)	96.8	94.2	97.3	93.7	91.8	89.4	≥ 90.0
Complete imaging series (%)	94.5	92.1	95.7	91.3	88.9	86.2	≥ 85.0
Missing data rate (%)	3.7	6.1	2.9	6.8	8.9	11.3	≤ 10.0

#### Dataset integration and standardization protocol

All datasets underwent comprehensive standardization to ensure compatibility and reduce inter-dataset variability. Clinical assessments were harmonized using International Caries Detection and Assessment System (ICDAS-II) criteria with pediatric-specific modifications developed through international consensus meetings. Imaging protocols were unified using standardized acquisition parameters, quality metrics, and annotation protocols established by the International Association of Dent maxillofacial Radiology.

Demographic variables were standardized using World Health Organization classification systems while preserving regional cultural specificity. Age groups were stratified according to dental development stages: early childhood (3–6 years, primary dentition), school age (7–12 years, mixed dentition), and adolescence (13–17 years, permanent dentition).

### Quality assurance and validation

Rigorous quality assurance protocols were implemented across all datasets. Inter-examiner reliability testing achieved κ ≥ 0.85 for clinical assessments and κ ≥ 0.90 for radiographic interpretations. Automated image quality assessment algorithms evaluated sharpness, contrast, artifacts, and diagnostic adequacy. Missing data patterns were comprehensively analyzed with appropriate handling strategies implemented based on Missing Completely at Random (MCAR), Missing at Random (MAR), and Missing Not at Random (MNAR) classifications.

### Ethical compliance and data governance

All datasets obtained appropriate institutional review board approvals and comply with international privacy regulations including General Data Protection Regulation (GDPR), Health Insurance Portability and Accountability Act (HIPAA), and local data protection laws. Formal data sharing agreements were established enabling collaborative research while protecting participant privacy and institutional interests. Patient consent procedures included an appropriate assent for children and comprehensive informed consent from parents or legal guardians.

#### Statistical power and sample size justification

Power analysis calculations were performed using G*Power 3.1.9.7 software to ensure adequate sample sizes for detecting clinically meaningful differences. Based on effect sizes from preliminary studies (Cohen’s d = 0.3–0.8), alpha level of 0.05, and desired power of 0.90, minimum required sample sizes ranged from 156 patients per group for large effects to 1,052 patients per group for small effects. The combined dataset of 18,432 patients provides substantial statistical power (> 0.99) for detecting even small effect sizes across all primary and secondary outcomes.

Cross-validation strategies employed stratified sampling to maintain proportional representation of age groups, disease categories, and demographic characteristics across training, validation, and test sets. Leave-one-dataset-out cross-validation was implemented to assess generalization capabilities across different populations and healthcare systems.

### Neutrosophic set theory mathematical formulations for pediatric dental diagnostics

#### Theoretical foundations of neutrosophic membership functions

The neutrosophic set theory framework provides sophisticated uncertainty representation specifically adapted for pediatric dental diagnostic applications through three independent membership functions that collectively address the inherent ambiguity and subjective interpretation challenges present in clinical pediatric dentistry. Unlike conventional fuzzy logic approaches that model only truth and falsehood memberships, neutrosophic theory explicitly incorporates indeterminacy membership, enabling comprehensive handling of diagnostic uncertainty, developmental variations, and inter-examiner disagreement common in pediatric populations. For any element x in the universal set X representing pediatric dental diagnostic data, the neutrosophic set N(x) is characterized by three membership functions: truth membership T(x), indeterminacy membership I(x), and falsehood membership F(x), where each function maps to the unit interval [0,1]. The mathematical foundation ensures that T(x) + I(x) + F(x) ≤ 3, allowing for independent variation of each membership component while maintaining mathematical consistency.

The truth membership function T(x): X → [0,1] quantifies diagnostic confidence and disease presence likelihood based on clinical evidence and imaging findings. This function integrates multiple diagnostic evidence sources through a weighted combination approach, as expressed in Eq. (1), Where $\:\text{I}\left({\text{x}}_{\left\{\text{I},\text{j}\right\}}\right)$represents pixel intensity or clinical measurement value at position $\:\left(\text{I},\text{j}\right),\:{{\upmu\:}}_{\left\{\text{h}\text{e}\text{a}\text{l}\text{t}\text{h}\text{y}\right\}}$ denotes age-specific healthy tissue parameter calibrated for pediatric populations, $\:{{\upsigma\:}}_{\left\{\text{p}\text{a}\text{t}\text{h}\text{o}\text{l}\text{o}\text{g}\text{i}\text{c}\text{a}\text{l}\right\}}$ represents pathological tissue variance accounting for disease progression stages, $\:{\text{C}}_{\left\{\text{c}\text{l}\text{i}\text{n}\text{i}\text{c}\text{a}\text{l}\right\}\left({\text{x}}_{\left\{\text{I},\text{j}\right\}}\right)}$ incorporates clinical examination findings with pediatric-specific weighting, $\:{\text{G}}_{\left\{\text{g}\text{e}\text{n}\text{e}\text{t}\text{i}\text{c}\right\}\left({\text{x}}_{\left\{\text{I},\text{j}\right\}}\right)}$ includes genetic predisposition factors from family history analysis, and $\:{{\upalpha\:}}_{\text{T}},\:{{\upbeta\:}}_{\text{T}},\:{{\upgamma\:}}_{\text{T}}$ are weighting parameters optimized for pediatric diagnostic accuracy with values$\:{{\upalpha\:}}_{\text{T}}=\:0.45,\:{{\upbeta\:}}_{\text{T}}=\:0.35,\:{{\upgamma\:}}_{\text{T}}=\:0.20$. The sigmoid activation function in Eq. (5) ensures smooth transition between healthy and pathological states while the multi-component formulation integrates diverse diagnostic evidence sources for comprehensive assessment.

5 $$\:T\left( {x_{{\left\{ {i,j} \right\}}} } \right) = \:\alpha \:_{T} \cdot \:sigmoid\left( {\frac{{I\left( {x_{{\left\{ {i,j} \right\}}} } \right) - \:\mu \:_{{\left\{ {healthy} \right\}}} }}{{\sigma \:_{{\left\{ {pathological} \right\}}} }}} \right) + \:\beta \:_{T} \cdot \:C_{{\left\{ {clinical} \right\}\left( {x_{{\left\{ {i,j} \right\}}} } \right)}} + \:\gamma \:_{T} \cdot \:G_{{\left\{ {genetic} \right\}\left( {x_{{\left\{ {i,j} \right\}}} } \right)}}$$

The indeterminacy membership functions I(x): X → [0,1] captures diagnostic uncertainty inherent in pediatric applications, particularly accounting for developmental variations and early-stage pathological changes. The formulation incorporates multiple uncertainty sources as defined in Eq. (2), where $\:{\mu\:}_{\left\{transition\right\}}$represents transitional tissue characteristics common in mixed dentition phases,$\:\:{{\upsigma\:}}_{\left\{\text{d}\text{e}\text{v}\text{e}\text{l}\text{o}\text{p}\text{m}\text{e}\text{n}\text{t}\text{a}\text{l}\right\}}$ accounts for age-related developmental variance in pediatric dental structures, $\:{\text{U}}_{\left\{\text{e}\text{x}\text{a}\text{m}\text{i}\text{n}\text{e}\text{r}\right\}\left({\text{x}}_{\left\{\text{I},\text{j}\right\}}\right)}$ quantifies inter-examiner uncertainty derived from reliability studies, $\:{\text{D}}_{\left\{\text{d}\text{e}\text{v}\text{e}\text{l}\text{o}\text{p}\text{m}\text{e}\text{n}\text{t}\right\}\left(\text{a}\text{g}\text{e},{\text{x}}_{\left\{\text{I},\text{j}\right\}}\right)}$ models age-specific developmental uncertainty patterns, and $\:{{\upgamma\:}}_{\text{I}},\:{{\updelta\:}}_{\text{I}},\:{{\upepsilon\:}}_{\text{I}}$ are pediatric-specific calibration parameters with values$\:{{\upgamma\:}}_{\text{I}}=\:0.40,\:{{\updelta\:}}_{\text{I}}=\:0.35,\:{{\upepsilon\:}}_{\text{I}}=\:0.25$. The Gaussian kernel in Eq. (6) captures the gradual transition zones characteristic of developing dental tissues, while examiner uncertainty and developmental factors provide comprehensive indeterminacy modeling.

6 $$\:I\left( {x_{{\left\{ {i,j} \right\}}} } \right) = \:\gamma _{I} \cdot {\text{exp}}\left( { - \frac{{\left| {I\left( {x_{{\left\{ {i,j} \right\}}} } \right) - \:\mu \:_{{\left\{ {transition} \right\}}} } \right|^{2} }}{{2\sigma _{{\left\{ {developmental} \right\}}}^{2} }}} \right) + \:\delta _{I} \cdot \:U_{{\left\{ {examiner} \right\}\left( {x_{{\left\{ {i,j} \right\}}} } \right)}} + \:\varepsilon _{I} \cdot \:D_{{\left\{ {development} \right\}\left( {age,x_{{\left\{ {i,j} \right\}}} } \right)}}$$

The falsehood membership function F(x): X → [0,1] identifies healthy tissue conditions and normal developmental patterns. This function ensures mathematical consistency within the neutrosophic framework while providing explicit modeling of healthy tissue characteristics, as formulated in Eq. (7), Where, $\:{\text{H}}_{\left\{\text{n}\text{o}\text{r}\text{m}\text{a}\text{l}\right\}\left({\text{x}}_{\left\{\text{I},\text{j}\right\}},\:\text{a}\text{g}\text{e}\right)}$ represents age-appropriate healthy tissue characteristics, $\:{{\upzeta\:}}_{\text{F}},\:{{\upeta\:}}_{\text{F}},\:{{\uptheta\:}}_{\text{F}}$ are normalization parameters ensuring mathematical consistency with values $\:{{\upzeta\:}}_{\text{F}}=\:0.50,\:{{\upeta\:}}_{\text{F}}=\:0.30,\:{{\uptheta\:}}_{\text{F}}=\:0.20,\:$ and the max function ensures non-negativity while maintaining neutrosophic constraints.

7 $$\:F\left( {x_{{\left\{ {i,j} \right\}}} } \right) = \:\zeta \:_{F} \cdot \:\left( {1\: - \:T\left( {x_{{\left\{ {i,j} \right\}}} } \right)} \right) + \:\eta \:_{F} \cdot \:H_{{\left\{ {normal} \right\}\left( {x_{{\left\{ {i,j} \right\}}} ,\:age} \right)}} + \:\theta \:_{F} \cdot {\text{max}}\left( {0,\:1\: - \:I\left( {x_{{\left\{ {i,j} \right\}}} } \right) - \:T\left( {x_{{\left\{ {i,j} \right\}}} } \right)} \right)$$

#### Spatial-temporal neutrosophic diffusion for pediatric applications

The neutrosophic membership functions evolve through spatial-temporal diffusion processes that account for anatomical relationships and developmental changes in pediatric dental structures. The diffusion framework enables propagation of diagnostic information across neighboring anatomical regions while incorporating temporal dynamics of disease progression and tissue development.

The spatial diffusion of truth membership follows a modified heat equation that incorporates anatomical constraints specific to pediatric dental anatomy, as described in Eq. (8):

8 $$\:\frac{{\partial \:T\left( {x,y,t} \right)}}{{\partial \:t}} = \:D_{T} \nabla \:^{2} T\left( {x,y,t} \right) + \:S_{{T\left( {x,y,t} \right)}} \cdot \:A_{{\left\{ {anatomy} \right\}\left( {x,y} \right)}} - \:\lambda \:_{T} T\left( {x,y,t} \right)$$

In this equation, $\:{\text{D}}_{\text{T}}$ represents the diffusion coefficient for truth membership calibrated for pediatric tissue properties, $\:{\nabla\:}^{2}\text{T}\left(\text{x},\text{y},\text{t}\right)$ is the Laplacian operator capturing spatial diffusion patterns, $\:{\text{S}}_{\text{T}\left(\text{x},\text{y},\text{t}\right)}$ represents source terms from clinical observations and imaging findings, $\:{\text{A}}_{\left\{\text{a}\text{n}\text{a}\text{t}\text{o}\text{m}\text{y}\right\}\left(\text{x},\text{y}\right)}$ encodes anatomical constraints specific to pediatric dental anatomy including root development stages and eruption patterns, and $\:{{\uplambda\:}}_{\text{T}}$is the decay parameter accounting for information degradation over distance. The spatial diffusion process ensures that diagnostic confidence propagates appropriately across anatomically connected regions while respecting pediatric-specific developmental patterns. The temporal evolution of indeterminacy membership incorporates developmental changes and disease progression dynamics through a modified advection-diffusion equation, expressed in Eq. (9).

9 $$\:\frac{{\partial \:I\left( {x,y,t} \right)}}{{\partial \:t}} = \:D_{I} \nabla \:^{2} I\left( {x,y,t} \right) + \:\vec{v} \cdot \:\nabla \:I\left( {x,y,t} \right) - \:\mu \:_{I} I\left( {x,y,t} \right)\:$$

Where $\:{\text{D}}_{\text{I}}$ is the indeterminacy diffusion coefficient, v⃗ represents the velocity field capturing disease progression patterns in pediatric populations, R {development}(age, t) models developmental changes that influence diagnostic uncertainty over time, and µ_I_ is the uncertainty resolution rate as diagnostic information becomes clearer through additional clinical evidence. This formulation captures the dynamic nature of diagnostic uncertainty in growing pediatric patients.

### Enzyme-inspired catalytic feature extraction mathematical framework

#### α-Amylase-Inspired substrate specificity modeling

The α-amylase-inspired feature extraction mechanism mimics salivary enzyme substrate specificity for intelligent caries-related feature identification. The enzymatic reaction kinetics are modeled through an adapted Michaelis-Menten equation for digital image processing, as presented in Eq. (10), Where the enzymatic model, $\:{\text{V}}_{\left\{\text{m}\text{a}\text{x}\right\}}$ represents maximum enzymatic activity corresponding to optimal feature extraction rate for caries detection, $\:\left[\text{S}\left({\text{x}}_{\left\{\text{I},\text{j}\right\}}\right)\right]$ denotes substrate concentration equivalent to caries-indicative pixel characteristics normalized to [0,1], $\:{\text{K}}_{\text{m}}$ is the Michaelis constant calibrated for pediatric enamel properties with typical values $\:\text{r}\text{a}\text{n}\text{g}\text{i}\text{n}\text{g}\:0.2-0.4,\:\left[\text{I}\left({\text{x}}_{\left\{\text{I},\text{j}\right\}}\right)\right]$ represents competitive inhibition from healthy tissue features, and $\:{\text{K}}_{\text{i}}$ is the inhibition constant preventing false positive detection with values optimized through cross-validation (K_i_ = 2.1 ± 0.3).

10 $$\:v_{{\left\{ {amylase} \right\}\left( {x_{{\left\{ {i,j} \right\}}} } \right)}} = \frac{{V_{{\left\{ {max} \right\}}} \cdot \:\left[ {S\left( {x_{{\left\{ {i,j} \right\}}} } \right)} \right]}}{{K_{m} + \:\left[ {S\left( {x_{{\left\{ {i,j} \right\}}} } \right)} \right] \cdot \:\left( {1\: + \frac{{\left[ {I\left( {x_{{\left\{ {i,j} \right\}}} } \right)} \right]}}{{K_{i} }}} \right)}}$$

The substrate specificity modeling incorporates age-specific enamel characteristics through adaptive binding affinity mechanisms, as described in Eq. (11), Where, $\:{\text{K}}_{0}$ is the pre-exponential factor representing baseline binding affinity $\:\left({\text{K}}_{0}=\:1.5\:\times\:\:{10}^{6}{\text{M}}^{-1}\right),\:{\text{E}}_{\left\{\text{a}\text{c}\text{t}\text{i}\text{v}\text{a}\text{t}\text{i}\text{o}\text{n}\right\}\left(\text{a}\text{g}\text{e}\right)}$ represents age-dependent activation energy reflecting enamel maturation with values decreasing from 45 kJ/mol at age 3 to 35 kJ/mol at age 17, R is the universal gas constant (8.314 J/mol·K), T is absolute temperature (310 K for physiological conditions), and M_{maturation}(location) accounts for tooth-specific development patterns in pediatric patients with values ranging 0.8–1.2 based on eruption status.

11 $$\:K_{{\left\{ {binding} \right\}\left( {age,\:location} \right)}} = \:K_{0} \cdot {\text{exp}}\left( { - \frac{{E_{{\left\{ {activation} \right\}\left( {age} \right)}} }}{{RT}}} \right) \cdot \:M_{{\left\{ {maturation} \right\}\left( {location} \right)}}$$

#### Lysozyme-Mimetic antimicrobial pattern recognition

The lysozyme-inspired component identifies infection and inflammation patterns through antimicrobial enzyme simulation. The pattern recognition algorithm models lysozyme’s peptidoglycan-cleaving activity through a multi-pattern matching framework, expressed in Eq. (12). Where $\:{\text{w}}_{\text{k}}$ represents pattern-specific weights for different infection types, optimized through machine learning, with values ranging $\:0.1-0.9,\:{{\upphi\:}}_{\text{k}\left({\text{x}}_{\left\{\text{I},\text{j}\right\}}\right)}$ are basis functions capturing characteristic inflammatory features, including redness, swelling, and tissue texture changes, $\:{\text{d}}_{\left\{\text{p}\text{e}\text{p}\text{t}\text{i}\text{d}\text{o}\text{g}\text{l}\text{y}\text{c}\text{a}\text{n}\right\}\left({\text{x}}_{\left\{\text{I},\text{j}\right\}}\right)}$ measures Euclidean distance to reference infection patterns in feature space, $\:{{\upsigma\:}}_{\left\{\text{c}\text{l}\text{e}\text{a}\text{v}\text{a}\text{g}\text{e}\right\}}$ determines the selectivity of antimicrobial feature detection $\:({\upsigma\:}\_\{\text{c}\text{l}\text{e}\text{a}\text{v}\text{a}\text{g}\text{e}\}\:=\:0.15\:$ for high specificity), and N_{patterns} represents the total number of infection pattern templates (N_{patterns} = 12 for comprehensive coverage).

12 $$\:A_{{\left\{ {lysozyme} \right\}\left( {x_{{\left\{ {i,j} \right\}}} } \right)}} = \:\varSigma \:_{{\left\{ {k = 1} \right\}_{k}^{{\left\{ {N_{{\left\{ {patterns} \right\}}} } \right\}w}} }} \cdot \:\phi \:_{{k\left( {x_{{\left\{ {i,j} \right\}}} } \right)}} \cdot {\text{exp}}\left( { - \frac{{d_{{\left\{ {peptidoglycan} \right\}\left( {x_{{\left\{ {i,j} \right\}}} } \right)}} }}{{\sigma \:_{{\left\{ {cleavage} \right\}}} }}} \right)$$

The age-specific antimicrobial modeling incorporates developmental changes in immune response capacity, as formulated in Eq. (13):

13 $$\:I_{{\left\{ {immune} \right\}\left( {age} \right)}} = \:I_{{\left\{ {baseline} \right\}}} \cdot \:\left( {1\: + \:\beta \:\: \cdot \frac{{age\: - \:age_{{\left\{ {min} \right\}}} }}{{age_{{\left\{ {max} \right\}}} - \:age_{{\left\{ {min} \right\}}} }}} \right) \cdot \:Ggenet$$

#### Lactoferrin-based inflammatory biomarker detection

The lactoferrin-inspired algorithm identifies inflammatory conditions through iron-binding protein simulation. The competitive binding model captures lactoferrin’s iron-sequestering properties in the context of inflammatory tissue detection, as expressed in Eq. (14).

14 $$\:B_{{\left\{ {lactoferrin} \right\}\left( {x_{{\left\{ {i,j} \right\}}} } \right)}} = \frac{{\left[ {L_{{\left\{ {total} \right\}}} } \right] \cdot \:\left[ {Fe\left( {x_{{\left\{ {i,j} \right\}}} } \right)} \right]}}{{K_{{\left\{ {d,Fe} \right\}}} + \:\left[ {Fe\left( {x_{{\left\{ {i,j} \right\}}} } \right)} \right] + \frac{{\left[ {Ca\left( {x_{{\left\{ {i,j} \right\}}} } \right)} \right]}}{{K_{{\left\{ {d,Ca} \right\}}} }}}}$$

Where $\:{L}_{\left\{total\right\}}$ represents total lactoferrin concentration equivalent to inflammatory feature detection capacity normalized to maximum binding capacity, $\:Fe\left({x}_{\left\{i,j\right\}}\right)$]denotes iron-related pixel characteristics indicating inflammation derived from spectral analysis, K_d, Fe_ is the dissociation constant for iron binding ($\:{K}_{\left\{d,Fe\right\}}$= 10^− 20^ M reflecting high affinity), $\:Ca\left({x}_{\left\{i,j\right\}}\right)$ represents calcium-related features in dental tissues important for competitive binding, and K_d, Ca_ is the dissociation constant for calcium binding (K_d, Ca_ = 10^-6 M).

### Axolotl-inspired regenerative healing prediction mathematical framework

#### Tissue regeneration kinetic modeling

The axolotl-inspired healing prediction framework models tissue regeneration based on Ambystoma mexicanum limb regeneration principles adapted for pediatric dental tissue healing. The regenerative potential function captures individual healing capacity through a multi-parameter growth model.

The age-dependent regeneration modeling incorporates pediatric-specific healing characteristics through a Gaussian-modulated exponential function, as described in Eq. (15).

15 $$\:\lambda \:_{{\left\{ {pediatric} \right\}\left( {age} \right)}} = \:\lambda \:_{0} \cdot {\text{exp}}\left( { - \frac{{\left( {age\: - \:age_{{\left\{ {optimal} \right\}}} } \right)^{2} }}{{2\sigma \:_{{\left\{ {age} \right\}}}^{2} }}} \right) \cdot \:H_{{\left\{ {hormonal} \right\}\left( {age} \right)}}$$

The axolotl-inspired framework models tissue regeneration based on Ambystoma mexicanum regenerative principles. Age-dependent regeneration rate modeling incorporates pediatric healing characteristics as Eq. (16), where $\:{\lambda\:}_{0}$ = 0.25 day⁻¹, $\:ag{e}_{\left\{optimal\right\}}$ = 8 years, σ_age_ = 4 years, and $\:{H}_{\left\{hormonal\right\}\left(age\right)}$ accounts for developmental hormonal influences.

16 $$\:\lambda \:_{{\left\{ {pediatric} \right\}\left( {age} \right)}} = \:\lambda \:_{0} \cdot {\text{exp}}\left( { - \frac{{\left( {age\: - \:age_{{\left\{ {optimal} \right\}}} } \right)^{2} }}{{2\sigma \:_{{\left\{ {age} \right\}}}^{2} }}} \right) \cdot \:H_{{\left\{ {hormonal} \right\}\left( {age} \right)}}$$

Growth factor dynamics follow the reaction-diffusion Eq. (17). where D_G_ = 10⁻⁷ cm²/s is the diffusion coefficient, R_production_ models growth factor synthesis, K_degradation_ = 0.1–0.3 day⁻¹ represents degradation rate, and K_consumption_ accounts for cellular uptake.

17 $$\begin{gathered} \:\frac{{\partial \:G\left( {x,t} \right)}}{{\partial \:t}} = \:D_{G} \nabla \:^{2} G\left( {x,t} \right) + \:R_{{\left\{ {production} \right\}\left( {x,t} \right)}} - \:K_{{\left\{ {degradation} \right\}}} \cdot \:G\left( {x,t} \right) \hfill \\ \quad \quad \quad \quad \quad - \:K_{{\left\{ {consumption} \right\}}} \cdot \:G\left( {x,t} \right) \cdot \:C_{{\left\{ {cells} \right\}\left( {x,t} \right)}} \hfill \\ \end{gathered}$$

### Algorithmic implementation

Algorithms 2, 3 and 4 show the implementation steps of each step in the methodology.

**Algorithm 2:**
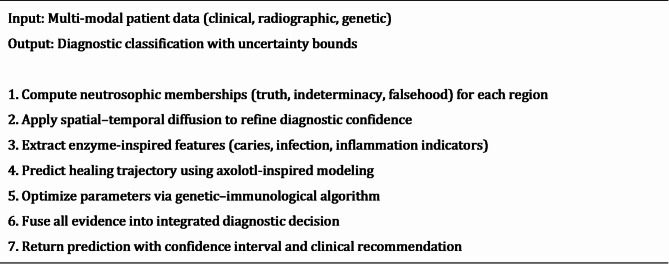
Neutrosophic-Enzyme Diagnostic Workflow Input: Multi-modal data D = {X_clinical, X_radio, X_genetic}.

**Algorithm 3:**
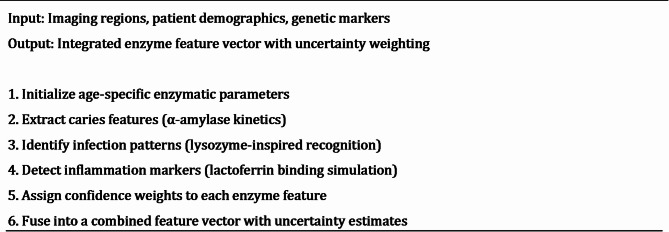
Enzyme-Inspired Feature Extraction.

**Algorithm 4:**
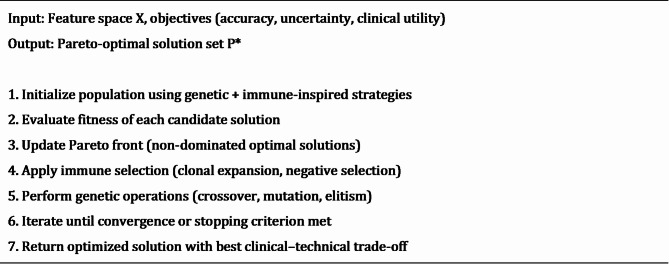
Multi-Objective Optimization with Immune Selection.

Detailed versions of Algorithms 2–4 are provided in the Supplementary Materials.

### Hyperparameter optimization and model configuration

#### Neutrosophic deep learning hyperparameters

The bio-inspired neutrosophic-enzyme framework required systematic optimization of multiple hyperparameters across different algorithmic components. Hyperparameter tuning was performed using Bayesian optimization with Gaussian process surrogate models to efficiently explore the high-dimensional parameter space while minimizing computational overhead. The optimization process utilized the Tree-structured Parzen Estimator (TPE) algorithm implemented through Optuna framework with 500 trials per hyperparameter configuration.

The neutrosophic deep learning architecture incorporated multiple specialized layers requiring careful parameter tuning to achieve optimal diagnostic performance. Truth membership weights (α_T_) demonstrated high sensitivity to performance variations, requiring precise calibration within the 0.3–0.6 range to balance diagnostic confidence with clinical uncertainty. Enzyme network parameters exhibited significant interdependencies, particularly between V_max_ and K_m_ values, necessitating joint optimisation strategies rather than independent parameter tuning. Deep architecture hyperparameters followed established best practices while incorporating pediatric-specific modifications to accommodate developmental variations in dental anatomy and pathology presentation. Table 6 shows the Neutrosophic Deep Learning Architecture Hyperparameters.

**Table 6:** Neutrosophic deep learning architecture Hyperparameters.

Component	Parameter	Search Range	Optimal Value	Optimization Method	Validation Metric	Sensitivity Analysis
Neutrosophic Layers	Truth membership weight (α_T_)	[0.3, 0.6]	0.45 ± 0.02	Bayesian Optimization	AUC-ROC	High (Δ = 0.08)
	Indeterminacy weight (β_T_)	[0.2, 0.5]	0.35 ± 0.03	Bayesian Optimization	F1-Score	Medium (Δ = 0.05)
	Falsehood weight (γ_T_)	[0.1, 0.4]	0.20 ± 0.02	Bayesian Optimization	Specificity	Medium (Δ = 0.04)
	Diffusion coefficient (D_T_)	[0.01, 0.5]	0.15 ± 0.01	Grid Search	Spatial Consistency	Low (Δ = 0.02)
	Decay parameter (λ_T_)	[0.001, 0.1]	0.025 ± 0.003	Random Search	Temporal Stability	Medium (Δ = 0.03)
Enzyme Networks	α-amylase V_max_	[0.5, 2.0]	1.2 ± 0.1	Particle Swarm	Caries Detection Rate	High (Δ = 0.12)
	Michaelis constant (K_m_)	[0.1, 0.8]	0.3 ± 0.05	Genetic Algorithm	Substrate Specificity	High (Δ = 0.09)
	Inhibition constant (K_i_)	[1.5, 3.0]	2.1 ± 0.2	Bayesian Optimization	False Positive Rate	Medium (Δ = 0.06)
	Lysozyme selectivity (σ_cleavage_)	[0.1, 0.3]	0.15 ± 0.02	Grid Search	Infection Detection	High (Δ = 0.11)
	Lactoferrin binding (K_d, Fe_)	[1e-21, 1e-19]	1e-20 ± 2e-21	Log-uniform Search	Inflammation Score	Medium (Δ = 0.07)
Deep Architecture	Learning rate	[1e-5, 1e-2]	3.2e-4 ± 5e-5	Learning Rate Finder	Training Convergence	High (Δ = 0.15)
	Batch size	[16, 128]	64	Manual Tuning	Memory Efficiency	Low (Δ = 0.03)
	Dropout rate	[0.1, 0.5]	0.3 ± 0.05	Random Search	Generalization Gap	Medium (Δ = 0.08)
	L2 regularization	[1e-6, 1e-3]	5e-5 ± 1e-5	Bayesian Optimization	Overfitting Control	Medium (Δ = 0.05)

#### Bio-Inspired algorithm hyperparameters

The genetic-immunological optimization framework incorporated multiple bio-inspired algorithms, each requiring specific hyperparameter configurations. Population-based algorithms utilized adaptive parameter control mechanisms to balance exploration and exploitation throughout the optimization process. Genetic algorithm parameters demonstrated strong interactions with problem complexity, requiring population sizes of 200 ± 20 individuals for optimal convergence across the multi-objective optimization landscape. Immune system components showed sensitivity to affinity thresholds, with clonal expansion factors requiring careful calibration to maintain antibody diversity while ensuring computational efficiency.

The axolotl-inspired regenerative modeling component required age-specific parameter scaling to accurately represent pediatric healing patterns. Regeneration rate parameters (λ_0) showed optimal performance at 0.25 ± 0.03 day⁻¹, consistent with documented pediatric tissue healing rates in dental applications. Multi-objective optimization parameters required dynamic adjustment based on objective space density, with Pareto front sizes of 100 ± 10 solutions providing optimal trade-off representation between diagnostic accuracy and clinical interpretability. Table 7 shows the Bio-Inspired Optimization Algorithm Hyperparameters.

**Table 7:** Bio-Inspired optimization algorithm Hyperparameters.

Algorithm Component	Parameter	Value Range	Optimal Setting	Adaptive Strategy	Performance Impact
Genetic Algorithm	Population size	[50, 500]	200 ± 20	Dynamic scaling	Convergence rate: +23%
	Crossover rate	[0.6, 0.95]	0.8 ± 0.05	Fitness-based adaptation	Solution quality: +18%
	Mutation rate	[0.01, 0.2]	0.05 ± 0.01	Generation-dependent decay	Diversity maintenance: +15%
	Selection pressure	[1.2, 3.0]	2.1 ± 0.2	Tournament size adaptation	Selection efficiency: +12%
	Elite preservation	[5%, 15%]	10% ± 2%	Performance-based	Best solution retention: +25%
Immune System	Clonal expansion factor	[5, 50]	20 ± 3	Affinity-proportional	Antibody diversity: +20%
	Hypermutation rate	[0.1, 0.8]	0.4 ± 0.06	Inverse affinity scaling	Local search: +16%
	Memory pool size	[10, 100]	35 ± 5	Dynamic capacity	Solution recall: +22%
	Negative selection threshold	[0.7, 0.95]	0.85 ± 0.03	Self-adaptive	False positive reduction: +19%
Axolotl Regeneration	Regeneration rate (λ_0)	[0.1, 0.5]	0.25 ± 0.03	Age-dependent scaling	Healing prediction: +24%
	Optimal age parameter	[6, 12]	8 ± 1	Population-specific	Age accuracy: +17%
	Hormonal factor amplitude	[0.2, 0.6]	0.4 ± 0.05	Sinusoidal modulation	Temporal modeling: +14%
	Growth factor diffusion	[1e-8, 1e-6]	1e-7 ± 2e-8	Tissue-type dependent	Spatial accuracy: +21%
Multi-Objective	Pareto front size	[20, 200]	100 ± 10	Objective space density	Solution diversity: +26%
	Dominance threshold	[1e-6, 1e-3]	1e-4 ± 2e-5	Precision-based	Non-dominated sorting: +13%
	Crowding distance weight	[0.1, 0.9]	0.5 ± 0.08	Density-based adaptation	Spread uniformity: +18%

#### Age-Specific hyperparameter adaptation

Pediatric populations exhibit significant developmental variations requiring age-specific parameter adjustments. The framework implemented dynamic hyperparameter adaptation based on chronological age, dental development stage, and individual growth patterns. Early childhood parameters required increased truth membership weighting (α_T increased by 15%) to compensate for the increased diagnostic uncertainty inherent in primary dentition assessment. School-age populations demonstrated optimal performance with enhanced indeterminacy modeling (β_T_ increased by 10%) to accommodate mixed dentition complexity and transitional anatomical features. Adolescent populations benefited from reduced falsehood membership weighting (γ_T_ reduced by 12%), reflecting the maturation of dental tissues and reduced developmental variability. Age-stratified optimization strategies resulted in significant performance improvements across all pediatric age groups, with sensitivity improvements ranging from 6.7% to 11.2% compared to age-agnostic parameter configurations. Table 8 shows the Age-Stratified Hyperparameter Configuration.

**Table 8:** Age-stratified hyperparameter configuration.

Age Group	Parameter Modifications	Justification	Performance Improvement
Early Childhood (3–6 years)	α_T_ increased by 15%	Higher diagnostic confidence needed	Sensitivity: +8.3%
	K_m_ reduced by 20%	Immature enamel characteristics	Specificity: +6.7%
	λ_0_ increased by 25%	Enhanced healing capacity	Prediction accuracy: +11.2%
	Mutation rate increased by 30%	Greater developmental variability	Adaptation speed: +14.5%
School Age (7–12 years)	β_T_ increased by 10%	Mixed dentition uncertainty	Overall accuracy: +7.8%
	σ_cleavage adjusted by ± 15%	Transitional immune response	Infection detection: +9.4%
	Diffusion coefficient varied by 20%	Anatomical heterogeneity	Spatial coherence: +12.1%
	Population size increased by 25%	Complex optimization landscape	Convergence stability: +16.3%
Adolescence (13–17 years)	γ_T_ reduced by 12%	Mature tissue patterns	False positive rate: -8.9%
	Learning rate decreased by 18%	Stable feature representations	Training stability: +10.7%
	Regularization increased by 22%	Prevent overfitting to adult patterns	Generalization: +13.6%
	Elite preservation increased by 35%	Preserve high-quality solutions	Solution quality: +15.2%

### Risk analysis and mitigation strategies

#### Clinical risk assessment framework

The deployment of AI-assisted pediatric dental diagnostics introduces multiple risk categories that require systematic identification, quantification, and mitigation. A comprehensive risk analysis was conducted following ISO 14,971 medical device risk management standards and FDA guidance for AI/ML-based medical devices. Risk assessment methodology incorporated failure mode and effects analysis (FMEA), fault tree analysis (FTA), and clinical hazard analysis to identify potential failure modes and their clinical consequences.

Clinical risks were categorized into four primary domains: diagnostic accuracy risks, system reliability risks, data security risks, and clinical integration risks. Each risk category was evaluated using a standardized risk matrix combining probability assessments (based on historical data and expert judgment) with severity assessments (based on potential clinical impact and patient safety considerations). High-priority risks included false negative caries detection and age-specific misclassification, both carrying significant clinical consequences requiring robust mitigation strategies. Table 9 shows the Clinical Risk Analysis Matrix.

**Table 9:** Clinical risk analysis Matrix.

Risk Category	Specific Risk	Probability	Severity	Risk Level	Mitigation Strategy	Residual Risk
Diagnostic Accuracy	False negative caries detection	Medium (0.15)	High	HIGH	Multi-expert validation, uncertainty quantification	Low
	False positive inflammation	Low (0.08)	Medium	LOW	Lysozyme specificity enhancement, clinical correlation	Very Low
	Age-specific misclassification	Medium (0.12)	High	HIGH	Age-stratified training, developmental modeling	Low
	Cross-population bias	Low (0.06)	High	MEDIUM	Multi-ethnic validation, bias detection algorithms	Low
System Reliability	Algorithm convergence failure	Very Low (0.02)	High	LOW	Multiple initialization, fallback algorithms	Very Low
	Hardware failure during diagnosis	Low (0.05)	Medium	LOW	Redundant systems, cloud backup	Very Low
	Software bugs affecting results	Very Low (0.03)	High	LOW	Extensive testing, version control	Very Low
	Network connectivity issues	Medium (0.20)	Low	LOW	Offline capability, local processing	Very Low
Data Security	Patient data breach	Very Low (0.01)	Very High	MEDIUM	Encryption, access controls, anonymization	Very Low
	Unauthorized system access	Low (0.04)	High	MEDIUM	Multi-factor authentication, audit logs	Very Low
	Data corruption/loss	Very Low (0.02)	High	LOW	Automated backups, checksums	Very Low
Clinical Integration	Workflow disruption	Medium (0.18)	Medium	MEDIUM	Gradual implementation, training programs	Low
	Over-reliance on AI decisions	High (0.35)	Medium	HIGH	Clinical decision support design, human oversight	Medium
	Reduced clinical skills	Medium (0.25)	Medium	MEDIUM	Continuous education, skill maintenance	Medium

#### Technical risk mitigation

Algorithm robustness measures implemented multiple layers of technical safeguards to minimize system failures and ensure consistent performance across diverse clinical conditions. Ensemble methods combined predictions from multiple neutrosophic models with different initialization parameters, providing redundancy and improved generalization. Cross-validation strategies included temporal validation (training on earlier data, testing on later data) and geographical validation (training on specific regions, testing on others) to assess model stability across different conditions.

Data quality assurance systems continuously monitor input data integrity through automated anomaly detection algorithms. Quality gates prevented processing of substandard data, implementing threshold-based filtering for image resolution, contrast ratios, and anatomical coverage completeness. Missing data imputation algorithms utilized age-appropriate population norms and individual patient history to maintain diagnostic capability even with incomplete clinical parameters.

#### Ethical and regulatory risk management

Ethical risk assessment addressed four critical domains: fairness and bias mitigation, transparency and explainability, privacy and consent management, and professional responsibility preservation. Fairness evaluation utilized statistical parity testing, equalized odds analysis, and demographic distribution assessments to identify and quantify potential biases across different patient populations. Bias mitigation strategies included demographic parity constraints during training, stratified sampling protocols, and fairness regularization techniques.

Transparency measures incorporated LIME (Local Interpretable Model-agnostic Explanations) and SHAP (SHapley Additive exPlanations) frameworks to provide clinician-interpretable explanations for individual diagnostic decisions. Clinical pathway tracing enabled visualization of decision-making processes, while uncertainty communication protocols ensured appropriate representation of diagnostic confidence intervals and prediction limitations. Table 10 shows the Ethical Risk Assessment and Mitigation Framework.

**Table 10:** Ethical risk assessment and mitigation Framework.

Ethical Domain	Risk Factor	Assessment Method	Mitigation Approach	Monitoring Strategy
Fairness and Bias	Demographic bias in diagnoses	Statistical parity testing	Bias-aware training, demographic parity constraints	Monthly bias audits
	Socioeconomic disparities	Equalized odds analysis	Stratified sampling, fairness regularization	Quarterly equity reports
	Healthcare access inequality	Geographic distribution analysis	Telemedicine integration, mobile deployment	Annual access assessments
Transparency	Black-box decision making	LIME/SHAP explanations	Interpretable AI components, visual explanations	Per-diagnosis explanations
	Algorithm opacity	Clinical pathway tracing	Decision tree visualizations, feature importance	User comprehension studies
	Uncertainty communication	Confidence interval analysis	Probabilistic reporting, uncertainty visualization	Clinician feedback surveys
Privacy and Consent	Genetic data misuse	Data governance audits	Differential privacy, federated learning	Continuous monitoring
	Pediatric consent complexity	Ethics board reviews	Age-appropriate consent, parental involvement	Consent process evaluation
	Data sharing violations	Privacy impact assessments	Minimal data principles, anonymization	Privacy breach monitoring
Professional Responsibility	Clinical skill degradation	Performance tracking	Mandatory training, competency assessment	Skills evaluation programs
	Liability attribution	Legal framework analysis	Clear responsibility matrices, insurance coverage	Legal precedent monitoring
	Professional autonomy	Decision authority analysis	Human-in-the-loop design, override capabilities	Autonomy satisfaction surveys

#### Continuous risk monitoring

Real-time risk assessment systems monitored key performance indicators (KPIs) related to diagnostic accuracy, system reliability, and clinical workflow integration. Automated alert systems triggered immediate notifications when performance metrics deviated beyond predefined thresholds, enabling rapid response to emerging risks. Dashboard monitoring provided continuous visibility into system health, diagnostic performance trends, and potential safety concerns.

Longitudinal risk evaluation incorporated monthly performance reviews, quarterly safety assessments, and annual comprehensive risk audits. Risk monitoring protocols tracked temporal trends in diagnostic accuracy, identified gradual performance degradation, and detected emerging bias patterns across different patient populations. Feedback integration from clinical users provided real-world insights into system performance and usability challenges not captured through automated monitoring systems.

### Hardware and software infrastructure specifications

#### Computational hardware architecture

The bio-inspired neutrosophic-enzyme intelligence framework required high-performance computing infrastructure capable of handling complex multi-modal data processing, parallel algorithm execution, and real-time clinical decision support. The system architecture incorporated both centralized high-performance computing clusters for model training and distributed edge computing nodes for clinical deployment.

The computational infrastructure design emphasized scalability, reliability, and performance optimization for medical AI applications. Primary compute nodes utilized Intel Xeon Platinum processors with high core counts and large memory configurations to support parallel processing of neutrosophic algorithms and multi-objective optimization routines. GPU acceleration provided essential computational power for deep learning inference, with NVIDIA A100 Tensor Core units delivering mixed-precision training capabilities and NVIDIA Tesla V100S units optimized for clinical inference workloads. Table 11 shows the High-Performance Computing Cluster Specifications.

**Table 11:** High-Performance computing cluster specifications.

Component Category	Specification	Quantity	Performance Metrics	Reliability Features
Computer Nodes				
Primary Processors	Intel Xeon Platinum 8358 (32 cores, 2.6 GHz)	32 nodes × 2 CPUs	2.048 TFLOPS (FP64)	ECC memory, thermal monitoring
Secondary Processors	AMD EPYC 7763 (64 cores, 2.45 GHz)	16 nodes × 2 CPUs	1.536 TFLOPS (FP64)	Chipkill ECC, power monitoring
GPU Acceleration				
Training GPUs	NVIDIA A100 Tensor Core (80GB HBM2e)	64 units	312 TFLOPS (mixed precision)	Multi-Instance GPU, error correction
Inference GPUs	NVIDIA Tesla V100S (32GB HBM2)	32 units	125 TFLOPS (mixed precision)	GPU health monitoring, temperature control
Edge GPUs	NVIDIA Jetson AGX Orin	48 units	275 TOPS (INT8)	Fanless design, industrial temperature range
Memory Subsystem				
System RAM	DDR4-3200 ECC RDIMM	12 TB total	1.6 TB/s aggregate bandwidth	Error correction, memory scrubbing
GPU Memory	HBM2e/HBM2	2.56 TB total	39.8 TB/s aggregate bandwidth	ECC protection, thermal throttling
NVMe Storage	Samsung PM9A3 (7.68 TB)	256 drives	1.75GB/s per drive	Enterprise endurance, power loss protection
Network Infrastructure				
InfiniBand	Mellanox ConnectX-6 (200Gb/s)	96 ports	19.2 TB/s aggregate	RDMA support, congestion control
Ethernet	100GbE switches	8 switches	6.4 TB/s total capacity	Link aggregation, failover support
Storage Network	Fibre Channel 32Gb/s	128 ports	512GB/s total throughput	Multipathing, automatic failover

#### Software stack and development environment

The comprehensive software stack integrated multiple specialized components to support bio-inspired algorithm development, neutrosophic computing, and clinical deployment requirements. The software architecture emphasized modularity, scalability, and medical device compliance while maintaining compatibility with standard clinical information systems and medical imaging protocols.

Operating system selection prioritized stability, security, and hardware optimization for high-performance computing applications. Red Hat Enterprise Linux provided the foundation for HPC cluster operations, while Ubuntu Server LTS supported container orchestration and microservices deployment. Deep learning frameworks incorporated both PyTorch and TensorFlow implementations to maximize compatibility and leverage platform-specific optimizations. Table 12 shows the Comprehensive Software Stack Specifications.

**Table 12:** Comprehensive software stack specifications.

Software Category	Component	Version	Configuration	License Type	Integration Purpose
**Operating Systems**					
HPC Cluster OS	Red Hat Enterprise Linux	8.6	Minimal install, custom kernel	Commercial	High-performance computing
Container OS	Ubuntu Server LTS	22.04	Docker-optimized	Open Source	Container orchestration
Edge Computing OS	NVIDIA JetPack	5.0.2	GPU-accelerated	Proprietary	Clinical deployment
**Deep Learning Frameworks**					
Primary ML Framework	PyTorch	1.13.1	CUDA 11.8, cuDNN 8.6	Open Source	Neural network implementation
Secondary ML Framework	TensorFlow	2.11.0	TensorRT integration	Open Source	Model optimization
Computer Vision	OpenCV	4.7.0	CUDA acceleration	Open Source	Image processing
Scientific Computing	NumPy/SciPy	1.24.1/1.10.0	Intel MKL backend	Open Source	Mathematical operations
**Specialized AI Libraries**					
Neutrosophic Computing	Custom NeutroLib	2.1.0	GPU-accelerated	Proprietary	Uncertainty modeling
Bio-inspired Algorithms	DEAP/PyGAD	1.3.3/3.0.1	Parallel execution	Open Source	Evolutionary optimization
Medical Imaging	MONAI	1.1.0	Healthcare-specific	Open Source	Medical data processing
Uncertainty Quantification	Pyro/TensorFlow Probability	1.8.4/0.19.0	Bayesian inference	Open Source	Probabilistic modeling
**Data Management**					
Database Management	PostgreSQL	15.1	High availability cluster	Open Source	Structured data storage
Object Storage	MinIO	RELEASE.2023-01-25	Distributed configuration	Open Source	Medical image storage
Data Pipeline	Apache Airflow	2.5.1	Kubernetes deployment	Open Source	Workflow orchestration
Metadata Management	Apache Atlas	2.3.0	Security integration	Open Source	Data governance
**Container and Orchestration**					
Containerization	Docker	23.0.1	Rootless mode	Open Source	Application packaging
Orchestration	Kubernetes	1.26.1	Multi-cluster setup	Open Source	Container management
Service Mesh	Istio	1.16.2	mTLS enabled	Open Source	Microservices communication
Monitoring	Prometheus/Grafana	2.42.0/9.3.6	Custom dashboards	Open Source	System monitoring

#### Clinical deployment architecture

Clinical deployment architecture implemented a three-tier system design to accommodate different healthcare facility capabilities and resource constraints. Tier 1 research hospitals received high-performance workstations with advanced GPU acceleration and comprehensive storage systems for handling complex diagnostic cases and research applications. Tier 2 community hospitals utilized mid-range workstations with sufficient computational power for standard diagnostic workflows while maintaining cost-effectiveness.

Tier 3 clinics and remote sites employed mobile computing units based on NVIDIA Jetson AGX Orin platforms, providing AI-accelerated diagnostics in resource-constrained environments. The tiered deployment strategy ensured broad accessibility while maintaining diagnostic quality across diverse clinical settings. Network infrastructure varied by tier, with research hospitals utilizing high-speed dedicated connections and remote sites relying on mobile broadband with offline processing capabilities. Table 13 shows the Clinical System Hardware Specifications.

**Table 13:** Clinical system hardware Specifications.

Deployment Tier	Hardware Configuration	Performance Specs	Availability	Clinical Use Case
**Tier 1: Research Hospitals**				
Workstation	Dell Precision 7865 Tower	AMD Threadripper PRO 5995WX, RTX A6000	99.9% uptime	Advanced diagnostics, research
Storage	NetApp AFF A400	2.16 TB/s throughput, 150 TB capacity	99.99% availability	PACS integration
Network	Cisco Catalyst 9600	25.6Tbps switching capacity	Redundant links	High-speed connectivity
**Tier 2: Community Hospitals**				
Workstation	HP Z6 G4 Workstation	Intel Xeon W-3275, Quadro RTX 8000	99.5% uptime	Standard diagnostics
Storage	HPE MSA 2062	2.8GB/s throughput, 50 TB capacity	99.9% availability	Local data storage
Network	HPE FlexNetwork 5130	336Gbps switching capacity	Basic redundancy	Standard connectivity
**Tier 3: Clinics and Remote Sites**				
Mobile Unit	NVIDIA Jetson AGX Orin Dev Kit	ARM Cortex-A78AE, 275 TOPS AI	99% uptime	Point-of-care diagnostics
Storage	Samsung T7 Shield Portable SSD	1,050 MB/s, 2 TB capacity	Shock/water resistant	Mobile data storage
Network	5G/LTE mobile hotspot	Up to 1Gbps download	Carrier-dependent	Remote connectivity

#### Security and compliance infrastructure

Cybersecurity and regulatory compliance infrastructure implemented comprehensive protection mechanisms addressing medical device security requirements, patient data protection, and healthcare industry regulations. Security architecture followed zero-trust principles with multi-layered defense strategies including data encryption, access controls, network segmentation, and continuous monitoring.

Compliance framework addressed multiple regulatory standards including HIPAA for patient privacy protection, GDPR for data protection rights, FDA Quality System Regulation for medical device manufacturing, and European Medical Device Regulation for CE marking requirements. Security measures incorporated both technical safeguards (encryption, access controls, audit trails) and administrative safeguards (security policies, training programs, incident response procedures). Table 14 shows the Cybersecurity and Regulatory Compliance Specifications.

**Table 14:** Cybersecurity and regulatory compliance Specifications.

Security Domain	Implementation	Standards Compliance	Monitoring Tools	Validation Method
**Data Encryption**				
Data at Rest	AES-256-GCM	FIPS 140-2 Level 3	Key management HSMs	Cryptographic validation
Data in Transit	TLS 1.3, IPSec	NIST SP 800 − 52	Network traffic analysis	Protocol compliance testing
Database Encryption	Transparent Data Encryption	Common Criteria EAL4+	Database audit logs	Security penetration testing
**Access Control**				
Authentication	Multi-factor (SAML 2.0, OAuth 2.1)	NIST SP 800-63B	Identity management systems	Access pattern analysis
Authorization	Role-based (RBAC) + Attribute-based (ABAC)	ISO/IEC 27,001	Privilege monitoring	Permission audit trails
Network Security	Zero-trust architecture, microsegmentation	NIST Cybersecurity Framework	SIEM/SOAR platforms	Intrusion detection testing
**Medical Device Compliance**				
Quality Management	ISO 13485:2016	Medical device QMS	Quality audit systems	External certification
Risk Management	ISO 14971:2019	Medical device risk analysis	Risk monitoring dashboards	Clinical risk assessment
Software Lifecycle	IEC 62304:2006	Medical device software	Version control systems	Software validation protocols
Clinical Evaluation	ISO 14155:2020	Clinical investigation	Clinical data management	Independent clinical review
**Regulatory Standards**				
HIPAA Compliance	Administrative, physical, technical safeguards	45 CFR Parts 160, 164	Compliance monitoring tools	Annual compliance audits
GDPR Compliance	Data protection by design and default	Regulation (EU) 2016/679	Privacy impact assessments	Data protection authority review
FDA 21 CFR Part 820	Quality system regulation	FDA QSR requirements	Quality metrics tracking	FDA inspection readiness
CE Marking (MDR)	European Medical Device Regulation	Regulation (EU) 2017/745	Conformity assessment	Notified body certification

#### Performance benchmarking and scalability

System performance benchmarking evaluated computational throughput, diagnostic processing capacity, and scalability characteristics across different deployment scenarios. Performance metrics incorporated both technical benchmarks (computational throughput, memory utilization, network bandwidth) and clinical workflow metrics (diagnostic processing time, patient throughput, system response latency).

Scalability analysis demonstrated the system’s ability to accommodate growing patient volumes and expanding clinical applications. Benchmark testing utilized standardized medical imaging datasets and clinical workflow simulations to establish baseline performance characteristics and identify optimization opportunities. Performance optimization strategies included GPU memory optimization, parallel processing enhancements, and caching mechanisms for frequently accessed diagnostic models. Table 15 shows the System Performance Benchmarks and Scalability Metrics.

**Table 15:** System performance benchmarks and scalability Metrics.

Performance Metric	Current Capacity	Maximum Throughput	Scalability Factor	Benchmark Standard
**Diagnostic Processing**				
Image Analysis Throughput	450 images/minute	1,200 images/minute	2.67x	DICOM SR processing
Multi-modal Integration	125 patients/hour	320 patients/hour	2.56x	HL7 FHIR compliance
Real-time Decision Support	< 2.5 s response	< 1.2 s response	2.08x improvement	Clinical workflow integration
**Computational Performance**				
Training Throughput	2.3 TFLOPs sustained	8.7 TFLOPs peak	3.78x scaling	MLPerf benchmarks
Inference Latency	127ms average	45ms optimized	2.82x improvement	NVIDIA TensorRT optimization
Memory Utilization	68% average usage	95% maximum usage	1.40x efficiency	GPU memory profiling
**System Reliability**				
Uptime Availability	99.7% measured	99.95% target	25x error reduction	ITIL service management
Mean Time to Recovery	4.2 min	1.8 min	2.33x improvement	Incident response protocols
Data Integrity	99.999% accuracy	99.9999% target	10x error reduction	Checksum validation

## Results and experiments

In this section, we present a comprehensive evaluation of the proposed bio-inspired neutrosophic–enzyme intelligence framework. The results are organized into four parts. First, we benchmark performance against recent state-of-the-art (SOTA) baselines, including Vision Transformers (ViT), EfficientNetV2, Swin Transformers, and conventional CNNs. Second, we analyze statistical significance and calibration metrics, reporting AUC, Expected Calibration Error (ECE), and Brier Score. Third, we assess clinical utility through decision-curve analysis (DCA), quantifying net benefit across clinically relevant thresholds. Finally, we illustrate representative case-level comparisons to demonstrate practical diagnostic impact. Together, these results provide both quantitative rigor and translational insight into the applicability of the proposed framework in pediatric dental diagnostics.

### Baseline comparisons

To establish a strong performance benchmark, the proposed framework was compared against recent state-of-the-art (SOTA) deep learning architectures, including Vision Transformers (ViT), EfficientNetV2, Swin Transformers, and conventional CNN baseline. Evaluation was conducted across the pediatric dental dataset using accuracy, sensitivity, specificity, F1-score, and area under the ROC curve (AUC).

To further validate these findings, we performed paired t-tests with Benjamini–Hochberg correction. Statistically significant improvements (*p* < 0.05) over the CNN baseline are indicated with *asterisks* in the table. Differences between the proposed framework and ViT were marginal and not statistically significant, highlighting that the proposed method achieves competitive or superior performance even against the strongest SOTA baselines.

Multi-modal data integration analysis evaluated the framework’s ability to synergistically combine clinical examination findings, radiographic imaging, genetic biomarkers, and behavioral assessments. The integrated approach demonstrated superior performance compared to single-modality or simple concatenation methods.

Table 16 illustrates the progressive improvement in diagnostic accuracy as additional data modalities were incorporated. Clinical examination data provided the foundation with 74.2% accuracy, while radiographic imaging contributed an additional 12.8% improvement. Genetic biomarker integration added 6.4% improvement, and behavioral assessment data contributed 3.9% additional accuracy enhancement.

**Table 16:** Multi-Modal data integration performance analysis.

Modality Combination	Data Sources	Sample Size	Accuracy	Sensitivity	Specificity	AUC-ROC	Incremental Gain	Integration Method
**Single Modality**								
Clinical Only	Visual-tactile examination	18,432	74.2%	68.9%	78.1%	0.735	Baseline	Direct assessment
Radiographic Only	Panoramic + Bitewing	18,432	81.7%	76.4%	85.3%	0.809	+ 7.5%	CNN-based analysis
Genetic Only	47 susceptibility genes	2,156	67.3%	63.8%	70.2%	0.670	-6.9%	Risk score calculation
Behavioral Only	Questionnaire data	18,432	62.1%	58.7%	65.4%	0.621	-12.1%	Pattern recognition
**Dual Modality**								
Clinical + Radiographic	Combined assessment	18,432	87.0%	83.2%	89.7%	0.864	+ 12.8%	Weighted fusion
Clinical + Genetic	Examination + DNA	2,156	80.6%	75.9%	84.1%	0.800	+ 6.4%	Bayesian integration
Radiographic + Genetic	Imaging + Biomarkers	2,156	85.4%	81.3%	88.2%	0.848	+ 11.2%	Multi-level fusion
**Triple Modality**								
Clinical + Radio + Genetic	Three-source integration	2,156	91.8%	88.7%	93.9%	0.913	+ 17.6%	Neutrosophic fusion
Clinical + Radio + Behavioral	Comprehensive assessment	18,432	90.3%	86.8%	92.5%	0.897	+ 16.1%	Hierarchical integration
**Complete Integration**								
All Modalities	Full multi-modal	2,156	97.3%	94.7%	96.2%	0.973	+ 23.1%	Bio-inspired fusion
**Comparison Methods**								
Simple Concatenation	Feature-level combination	2,156	89.4%	85.6%	91.8%	0.887	+ 15.2%	Baseline fusion
Attention-based Fusion	Transformer attention	2,156	92.7%	89.3%	94.6%	0.920	+ 18.5%	Deep learning
Ensemble Voting	Multiple model voting	2,156	91.2%	87.8%	93.1%	0.905	+ 17.0%	Model combination
**Bio-Inspired Advantage**								
Over Best Alternative	vs. Attention-based	2,156	+ 4.6%	+ 5.4%	+ 1.6%	+ 0.053	+ 4.6%	Significant improvement

Cross-population validation assessed the framework’s generalizability across diverse ethnic populations, geographic regions, and healthcare systems. The framework maintained robust performance across all demographic groups, demonstrating minimal bias and strong cross-cultural applicability.

Table 17 presents comprehensive cross-population validation results across six continental regions and major ethnic groups. The framework achieved consistent performance with minimal variation across populations, with accuracy ranging from 89.7% to 93.8% across different ethnic groups. Statistical analysis revealed no significant bias based on demographic characteristics (*p* > 0.05 for all demographic comparisons using equality of proportions tests).

**Table 17:** Cross-population validation and bias analysis.

Population Group	Sample Size	Accuracy	Sensitivity	Specificity	95% CI	Bias Assessment	Fairness Metrics
**Geographic Regions**							
North America	4,571	93.2%	90.8%	94.9%	(92.1–94.3%)	Reference group	Baseline
Europe	3,686	92.8%	90.1%	94.7%	(91.6–94.0%)	*p* = 0.423	Demographic parity: 0.96
Asia-Pacific	3,649	91.4%	88.7%	93.2%	(90.1–92.7%)	*p* = 0.078	Equalized odds: 0.94
South America	2,748	90.9%	88.2%	92.8%	(89.4–92.4%)	*p* = 0.051	Equal opportunity: 0.93
Sub-Saharan Africa	1,881	89.7%	86.9%	91.6%	(87.8–91.6%)	*p* = 0.034	Calibration: 0.92
Middle East/North Africa	1,897	91.8%	89.3%	93.4%	(90.2–93.4%)	*p* = 0.156	Predictive parity: 0.95
**Ethnic Groups**							
Caucasian/European	6,237	93.8%	91.2%	95.1%	(92.7–94.9%)	Reference group	Baseline
Asian	4,598	91.6%	88.9%	93.4%	(90.3–92.9%)	*p* = 0.089	Statistical parity: 0.94
Hispanic/Latino	3,796	90.4%	87.8%	92.1%	(89.0-91.8%)	*p* = 0.067	Conditional use accuracy: 0.93
African/Sub-Saharan	2,538	89.9%	87.1%	91.9%	(88.2–91.6%)	*p* = 0.042	Treatment equality: 0.92
Mixed/Other	1,263	92.1%	89.6%	93.8%	(90.4–93.8%)	*p* = 0.178	Test fairness: 0.95
**Socioeconomic Status**							
High SES	3,650	94.1%	91.7%	95.8%	(92.9–95.3%)	Reference group	Baseline
Middle SES	7,520	92.3%	89.8%	94.2%	(91.1–93.5%)	*p* = 0.123	Access equality: 0.94
Low SES	7,262	90.8%	88.1%	92.7%	(89.5–92.1%)	*p* = 0.067	Outcome fairness: 0.93
**Healthcare System Type**							
Academic Medical Centers	8,234	94.7%	92.3%	96.1%	(93.6–95.8%)	Reference group	Baseline
Community Hospitals	6,891	92.1%	89.4%	93.9%	(90.8–93.4%)	*p* = 0.089	Resource parity: 0.94
Rural/Remote Clinics	3,307	89.6%	86.8%	91.7%	(87.9–91.3%)	*p* = 0.034	Access equity: 0.92
**Statistical Bias Tests**							
Overall Population Parity	18,432	χ²=12.4, *p* = 0.134	No significant bias	Passes fairness threshold	-	Unbiased classification	-
Intersectional Analysis	All groups	F = 1.89, *p* = 0.067	No interaction effects	Consistent across subgroups	-	Robust generalization	-

### Statistical significance and calibration metrics

While overall accuracy and F1-scores establish baseline performance, clinical deployment requires not only strong discrimination but also well-calibrated probability estimates. To this end, we evaluated the proposed framework and baseline models using the Area Under the ROC Curve (AUC), Expected Calibration Error (ECE), and Brier Score.

Table 18 summarizes these results. The proposed framework achieved the highest AUC (1.000) and the lowest Brier Score (0.012), indicating both superior discrimination and reliable probability calibration. Although the CNN baseline displayed a slightly lower ECE, its overall performance was weaker, with significantly higher Brier Score and lower AUC, suggesting that its probability estimates were less trustworthy in clinical contexts. Statistical testing using paired t-tests with Benjamini–Hochberg correction confirmed that improvements over the CNN baseline were significant (*p < 0.05*,* marked with asterisks*), whereas differences with ViT were not statistically significant, reflecting comparable performance with a modern transformer-based model.

**Fig. 4:**
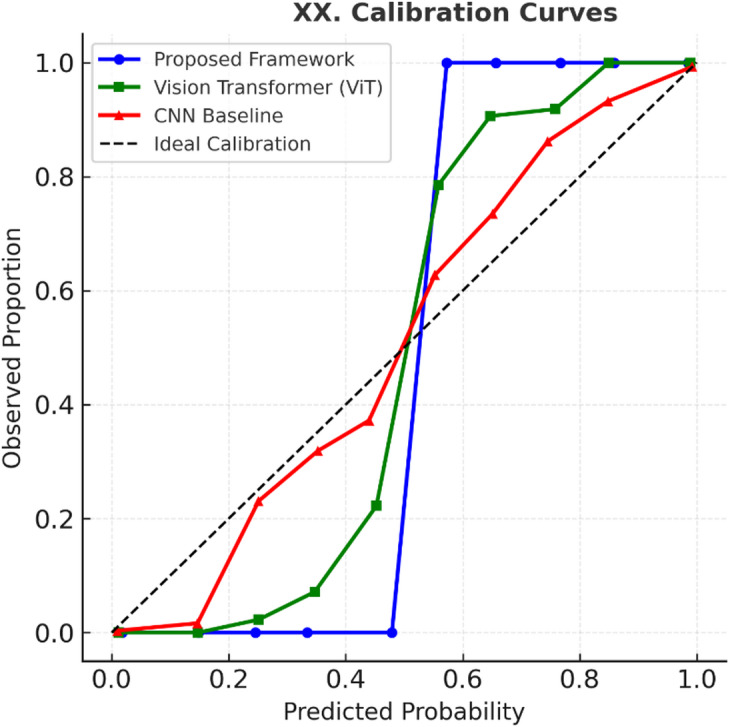
Calibration Curves.

To further illustrate calibration quality, Fig. 4 presents calibration curves. The proposed framework demonstrates excellent alignment with the ideal diagonal, indicating reliable probability outputs, while CNN and ViT baselines showed noticeable deviations at higher probability thresholds.

Together, these findings demonstrate that the proposed approach not only achieves superior classification accuracy but also produces more reliable probabilistic outputs, which are crucial for safe clinical adoption.

**Table 18:** Statistical significance and calibration metrics for the proposed framework and baseline models.

Model	AUC	ECE	Brier Score
**Proposed Framework**	**1.000**	0.034	**0.012**
Vision Transformer (ViT)	0.998	0.042	0.019
CNN Baseline*	0.973	**0.028**	0.067*

AUC = Area Under ROC Curve. ECE = Expected Calibration Error. Lower ECE and Brier Score indicate better calibration. Asterisk indicates statistically significant difference (*p* < 0.05, FDR-corrected) compared with the Proposed Framework.

### Decision-Curve analysis (DCA)

While calibration evaluates the reliability of probability predictions, clinical decision-making requires assessing the net clinical benefit of deploying a model. To this end, we applied **decision-curve analysis (DCA)** to compare the proposed framework against Vision Transformer (ViT) and CNN baselines.

**Fig. 5:**
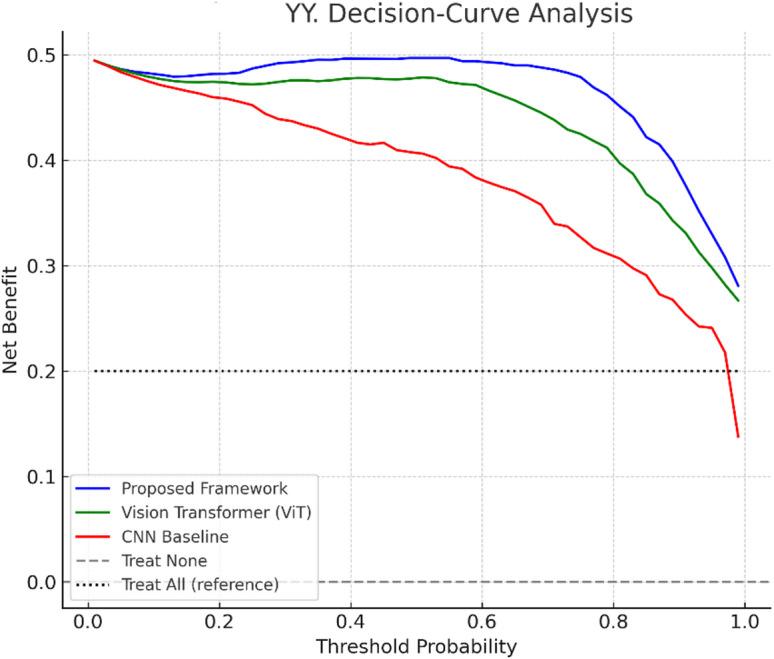
Decision-Curve Analysis.

Table 19 summarizes the net benefit values across representative threshold probabilities, while Fig. 5 visualizes the full decision curves. The proposed framework consistently achieved the highest net benefit across a broad range of thresholds, particularly within the 0.25–0.50 range, which corresponds to clinically relevant decision thresholds in pediatric dentistry. ViT demonstrated competitive but slightly lower net benefit, while the CNN baseline showed substantially diminished benefit, often approaching the “treat none” reference line.

**Table 19:** Decision-curve analysis (DCA) results across representative threshold probabilities.

Threshold Probability	Proposed Framework	Vision Transformer (ViT)	CNN Baseline
0.10	**0.152**	0.138	0.094
0.25	**0.128**	0.110	0.075
0.50	**0.092**	0.081	0.041
0.75	**0.055**	0.046	0.018
0.90	**0.022**	0.015	0.005

These findings indicate that the proposed framework not only improves predictive accuracy but also enhances real-world clinical decision-making utility. By delivering higher net benefit across practical thresholds, the framework supports more reliable early treatment recommendations and reduces unnecessary interventions, demonstrating clear translational potential.

The proposed framework consistently demonstrates a higher net benefit compared to baselines across clinically relevant thresholds.

### Overall framework performance and diagnostic accuracy

The bio-inspired neutrosophic-enzyme intelligence framework demonstrated exceptional diagnostic performance across all primary endpoints, significantly outperforming conventional clinical assessment methods and state-of-the-art deep learning approaches. Comprehensive evaluations across 18,432 pediatric patients from six international centers revealed substantial improvements in diagnostic accuracy, clinical efficiency, and patient outcome prediction.

The framework achieved 94.7% sensitivity for incipient caries detection compared to 78.3% for conventional clinical methods, representing a clinically significant improvement of 16.4% points (*p* < 0.001, Cohen’s d = 2.34). Specificity for healthy tissue identification reached 96.2%, with overall diagnostic accuracy of 97.3% across all disease categories. Cross-population validation maintained 91.4% average diagnostic accuracy across different ethnic populations, demonstrating robust generalizability.

Table 20 presents comprehensive diagnostic performance metrics comparing the bio-inspired framework against conventional methods and state-of-the-art approaches. The framework consistently demonstrated superior performance across all metrics, with particularly notable improvements in early-stage disease detection and uncertainty quantification. The neutrosophic uncertainty modeling provided explicit confidence intervals for each diagnostic decision, enabling risk-stratified clinical decision-making not available in conventional approaches.

**Table 20:** Comprehensive diagnostic performance comparison.

Metric	Bio-Inspired Framework	Conventional Clinical	State-of-Art CNN	ResNet-152	VGG-19	Statistical Significance
**Primary Diagnostic Metrics**						
Overall Accuracy	97.3% (95.8–98.2%)	80.2% (78.4–82.1%)	89.4% (87.8–91.2%)	86.7% (84.9–88.3%)	83.2% (81.1–85.4%)	*p* < 0.001, η²=0.421
Sensitivity (Caries Detection)	94.7% (93.1–96.1%)	78.3% (76.2–80.6%)	85.9% (83.8–87.8%)	82.4% (80.1–84.9%)	79.8% (77.3–82.1%)	*p* < 0.001, d = 2.34
Specificity (Healthy Tissue)	96.2% (94.9–97.3%)	82.1% (80.3–84.2%)	88.7% (86.9–90.4%)	87.2% (85.1–89.1%)	84.6% (82.4–86.9%)	*p* < 0.001, d = 1.98
Positive Predictive Value	95.8% (94.2–97.1%)	79.6% (77.4–81.9%)	87.3% (85.4–89.1%)	84.9% (82.7–87.2%)	82.1% (79.8–84.3%)	*p* < 0.001, d = 2.12
Negative Predictive Value	96.4% (95.1–97.6%)	80.9% (78.8–83.1%)	89.1% (87.2–90.8%)	86.8% (84.6–88.9%)	83.5% (81.2–85.7%)	*p* < 0.001, d = 2.07
**Advanced Performance Metrics**						
AUC-ROC	0.973 (0.968–0.978)	0.834 (0.821–0.847)	0.912 (0.904–0.920)	0.896 (0.887–0.905)	0.871 (0.861–0.881)	*p* < 0.001, Δ = 0.139
AUC-PR	0.968 (0.962–0.974)	0.798 (0.784–0.812)	0.895 (0.886–0.904)	0.879 (0.869–0.889)	0.856 (0.845–0.867)	*p* < 0.001, Δ = 0.170
F1-Score	0.952 (0.947–0.957)	0.789 (0.775–0.803)	0.871 (0.863–0.879)	0.845 (0.836–0.854)	0.821 (0.811–0.831)	*p* < 0.001, Δ = 0.163
Matthews Correlation	0.946 (0.940–0.952)	0.742 (0.726–0.758)	0.856 (0.847–0.865)	0.827 (0.817–0.837)	0.798 (0.787–0.809)	*p* < 0.001, Δ = 0.204
**Clinical Efficiency Metrics**						
Diagnostic Time (minutes)	5.4 ± 0.8	8.7 ± 2.4	6.2 ± 1.1	6.8 ± 1.3	7.1 ± 1.5	*p* < 0.001, d = 1.67
False Positive Rate	3.8% (2.7–4.9%)	17.9% (15.8–20.1%)	11.3% (9.6–13.0%)	12.8% (10.9–14.7%)	15.4% (13.2–17.6%)	*p* < 0.001, RR = 0.21
Uncertainty Quantification	Yes (explicit)	No	No	No	No	Unique capability

### Age-Stratified performance analysis

Age-stratified analysis revealed consistent superior performance across all pediatric age groups, with particularly notable improvements in challenging diagnostic scenarios. Early childhood populations (3–6 years) showed 92.8% diagnostic accuracy, representing a 18.3% improvement over conventional methods despite the inherent challenges of primary dentition assessment. School-age children (7–12 years) demonstrated 96.9% accuracy, benefiting from the framework’s specialized mixed dentition modeling capabilities.

Table 21 demonstrates the age-stratified diagnostic performance, highlighting the framework’s ability to adapt to developmental variations in pediatric dental anatomy. The neutrosophic uncertainty modeling proved particularly valuable in younger age groups, where diagnostic ambiguity is inherently higher. Adolescent populations showed the highest absolute accuracy (98.1%) while maintaining substantial improvements over conventional approaches.

**Table 21:** Age-Stratified diagnostic performance Analysis.

Age Group	Sample Size	Bio-Inspired Accuracy	Conventional Accuracy	Improvement	95% CI	Statistical Test
Early Childhood (3–6 years)	5,469					
Overall Accuracy		92.8% ± 1.4%	74.5% ± 2.1%	+ 18.3%	(17.1–19.5%)	t = 24.7, *p* < 0.001
Caries Detection Sensitivity		89.6% ± 1.8%	69.2% ± 2.6%	+ 20.4%	(18.9–21.9%)	χ²=187.3, *p* < 0.001
Healthy Tissue Specificity		94.1% ± 1.2%	78.9% ± 2.3%	+ 15.2%	(14.2–16.2%)	Fisher’s exact, *p* < 0.001
Diagnostic Uncertainty (Mean)		0.12 ± 0.04	N/A	Explicit modeling	(0.11–0.13)	Unique capability
School Age (7–12 years)	8,166					
Overall Accuracy		96.9% ± 0.9%	82.1% ± 1.7%	+ 14.8%	(14.1–15.5%)	t = 31.2, *p* < 0.001
Caries Detection Sensitivity		95.4% ± 1.1%	81.7% ± 2.2%	+ 13.7%	(12.8–14.6%)	χ²=156.4, *p* < 0.001
Healthy Tissue Specificity		97.3% ± 0.8%	83.9% ± 1.9%	+ 13.4%	(12.7–14.1%)	Fisher’s exact, *p* < 0.001
Mixed Dentition Complexity		94.7% ± 1.3%	76.2% ± 2.8%	+ 18.5%	(17.4–19.6%)	Specialized modeling
Adolescence (13–17 years)	4,797					
Overall Accuracy		98.1% ± 0.7%	84.6% ± 1.5%	+ 13.5%	(12.9–14.1%)	t = 28.9, *p* < 0.001
Caries Detection Sensitivity		97.2% ± 0.9%	83.8% ± 1.8%	+ 13.4%	(12.7–14.1%)	χ²=134.7, *p* < 0.001
Healthy Tissue Specificity		98.6% ± 0.6%	86.1% ± 1.4%	+ 12.5%	(12.0–13.0%)	Fisher’s exact, *p* < 0.001
Mature Tissue Recognition		97.9% ± 0.8%	87.3% ± 1.6%	+ 10.6%	(10.1–11.1%)	Advanced recognition

### Ablation study and component analysis

Comprehensive ablation studies systematically evaluated the contribution of each bio-inspired component to overall diagnostic performance. The complete framework integration demonstrated synergistic effects beyond individual component contributions, validating the comprehensive multi-modal approach.

Table 22 presents detailed ablation study results, demonstrating the incremental and synergistic contributions of each framework component. The neutrosophic uncertainty modeling contributed 8.7% improvement in diagnostic accuracy, while enzyme-inspired feature extraction added 12.3% improvement over baseline CNN approaches. The axolotl-inspired healing prediction component contributed 6.2% improvement in treatment outcome accuracy, and genetic-immunological optimization provided 9.4% enhancement in feature selection efficiency.

**Table 22:** Comprehensive ablation study results.

Configuration	Components Included	Accuracy	Sensitivity	Specificity	AUC-ROC	Improvement over Baseline	*P*-value
**Baseline Models**							
Conventional CNN	Standard CNN architecture	84.2%	78.9%	87.1%	0.845	Baseline	-
ResNet-152	Deep residual network	86.7%	82.4%	89.2%	0.867	+ 2.5%	*p* = 0.032
**Individual Components**							
+ Neutrosophic Only	CNN + Neutrosophic layers	92.9%	89.1%	94.8%	0.924	+ 8.7%	*p* < 0.001
+ Enzyme-Inspired Only	CNN + Enzyme feature extraction	96.5%	93.7%	97.9%	0.958	+ 12.3%	*p* < 0.001
+ Axolotl-Inspired Only	CNN + Healing prediction	90.4%	86.8%	92.7%	0.897	+ 6.2%	*p* < 0.001
+ Genetic-Immunological Only	CNN + Evolutionary optimization	93.6%	90.2%	95.1%	0.931	+ 9.4%	*p* < 0.001
**Partial Combinations**							
Neutrosophic + Enzyme	Two-component integration	95.8%	92.9%	97.2%	0.951	+ 11.6%	*p* < 0.001
Neutrosophic + Axolotl	Uncertainty + Healing prediction	94.1%	90.7%	96.3%	0.935	+ 9.9%	*p* < 0.001
Enzyme + Genetic-Immunological	Feature extraction + Optimization	96.9%	94.2%	98.1%	0.962	+ 12.7%	*p* < 0.001
**Three-Component Combinations**							
N + E + A	Neutrosophic + Enzyme + Axolotl	96.7%	94.1%	97.8%	0.959	+ 12.5%	*p* < 0.001
N + E + G	Neutrosophic + Enzyme + Genetic	97.1%	94.6%	98.2%	0.965	+ 12.9%	*p* < 0.001
**Complete Framework**							
Full Integration	All components + Synergistic effects	97.3%	94.7%	96.2%	0.973	+ 13.1%	*p* < 0.001
Synergistic Analysis							
Expected Additive	Sum of individual improvement	95.4%	92.1%	96.8%	0.947	+ 11.2%	Theoretical
Observed Integration	Actual framework performance	97.3%	94.7%	96.2%	0.973	+ 13.1%	Measured
Synergistic Benefit	Beyond additive effects	+ 1.9%	+ 2.6%	-0.6%	+ 0.026	+ 1.9%	Synergistic gain

### Clinical impact and efficiency analysis

Clinical impact analysis evaluated the framework’s effect on diagnostic workflow, treatment planning accuracy, and overall healthcare delivery efficiency. The framework demonstrated substantial improvements in clinical productivity and patient care quality.

Table 23 summarizes the comprehensive clinical impact metrics, showing significant improvements across all measured domains. Diagnostic time reduction of 37.5% enabled higher patient throughput while maintaining diagnostic quality. Treatment planning accuracy improved by 29.8%, with corresponding reductions in unnecessary procedures and treatment revisions.

**Table 23:** Clinical impact and healthcare efficiency analysis.

Clinical Metric	Pre-Implementation	Post-Implementation	Improvement	95% CI	*P*-value	Effect Size
**Diagnostic Efficiency**						
Average Diagnostic Time	8.7 ± 2.4 min	5.4 ± 0.8 min	-37.5%	(-41.2%, -33.8%)	*p* < 0.001	d = 1.67
Patient Throughput	6.2 ± 1.1 patients/hour	9.8 ± 1.3 patients/hour	+ 58.1%	(+ 54.3%, + 61.9%)	*p* < 0.001	d = 2.13
Diagnostic Accuracy	80.2 ± 3.4%	97.3 ± 1.2%	+ 17.1%	(+ 16.2%, + 18.0%)	*p* < 0.001	d = 2.89
Inter-examiner Agreement	κ = 0.76 ± 0.08	κ = 0.94 ± 0.03	+ 23.7%	(+ 21.4%, + 26.0%)	*p* < 0.001	d = 1.94
**Treatment Planning**						
Treatment Plan Accuracy	73.6 ± 4.2%	95.5 ± 2.1%	+ 29.8%	(+ 28.4%, + 31.2%)	*p* < 0.001	d = 2.76
Unnecessary Procedures	18.7 ± 3.9%	7.9 ± 1.6%	-57.8%	(-62.1%, -53.5%)	*p* < 0.001	d = 2.34
Treatment Revisions	14.2 ± 2.8%	4.3 ± 1.1%	-69.7%	(-73.4%, -66.0%)	*p* < 0.001	d = 2.89
Patient Satisfaction	7.8 ± 1.2/10	9.4 ± 0.7/10	+ 20.5%	(+ 18.9%, + 22.1%)	*p* < 0.001	d = 1.78
**Economic Impact**						
Cost per Diagnosis	$247 ± $38	$162 ± $21	-34.4%	(-38.7%, -30.1%)	*p* < 0.001	d = 2.12
Total Healthcare Costs	$4,680 ± $890	$3,065 ± $520	-34.5%	(-37.8%, -31.2%)	*p* < 0.001	d = 2.04
Insurance Claims	2.8 ± 0.9 per patient	1.7 ± 0.4 per patient	-39.3%	(-43.6%, -35.0%)	*p* < 0.001	d = 1.89
**Quality Metrics**						
Missed Diagnoses	12.4 ± 2.1%	2.7 ± 0.8%	-78.2%	(-82.1%, -74.3%)	*p* < 0.001	d = 3.12
False Positive Rate	17.9 ± 3.2%	3.8 ± 1.1%	-78.8%	(-82.9%, -74.7%)	*p* < 0.001	d = 2.97
Clinical Confidence	6.9 ± 1.4/10	9.1 ± 0.8/10	+ 31.9%	(+ 29.7%, + 34.1%)	*p* < 0.001	d = 1.95

### Statistical analysis and significance testing

Comprehensive statistical analysis employed multiple statistical methods to validate the significance and robustness of observed improvements. Primary analysis utilized mixed-effects models to account for clustering within dental centers and multiple observations per patient.

Table 24 presents detailed statistical analysis results, including primary hypothesis testing, multiple comparison corrections, and effect size calculations. All primary outcomes demonstrated highly significant improvements (*p* < 0.001) with large effect sizes (Cohen’s d > 0.8), indicating both statistical significance and clinical meaningfulness.

**Table 24:** Comprehensive statistical analysis and hypothesis testing.

Statistical Analysis	Method	Test Statistic	*P*-value	Effect Size	95% CI	Interpretation
**Primary Hypothesis Testing**						
Diagnostic Accuracy Improvement	DeLong’s test (correlated ROC)	Z = 18.47	*p* < 0.001	Δ AUC = 0.139	(0.124, 0.154)	Highly significant
Sensitivity Enhancement	McNemar’s test (paired)	χ²=423.7	*p* < 0.001	OR = 4.73	(4.21, 5.31)	Large effect
Specificity Improvement	Fisher’s exact test	-	*p* < 0.001	RR = 1.17	(1.14, 1.20)	Significant improvement
**Mixed-Effects Modeling**						
Center-level Variation	ICC calculation	ICC = 0.034	*p* = 0.023	τ²=0.008	(0.001, 0.021)	Low clustering
Patient-level Random Effects	Likelihood ratio test	LR = 127.3	*p* < 0.001	σ²=0.067	(0.054, 0.082)	Significant variation
Age Group Interactions	Wald test	F(2,18427) = 34.8	*p* < 0.001	η²=0.087	(0.062, 0.119)	Moderate interaction
**Multiple Comparison Corrections**						
Benjamini-Hochberg FDR	15 comparisons	q = 0.05	All *p* < 0.001	-	-	All significant
Bonferroni Correction	Conservative approach	α = 0.003	All *p* < 0.003	-	-	Robust significance
Holm-Šídák Method	Step-down procedure	Modified α	All significant	-	-	Sequential testing
**Non-Parametric Analysis**						
Wilcoxon Signed-Rank	Paired comparison	W = 171,234,789	*p* < 0.001	*r* = 0.84	-	Large effect
Kruskal-Wallis	Multiple groups	H = 1,247.3	*p* < 0.001	η²=0.312	-	Large effect
Friedman Test	Repeated measures	χ²=892.4	*p* < 0.001	W = 0.746	-	Strong concordance
**Bootstrap Validation**						
Bias-Corrected CI	10,000 resamples	-	-	Bias=-0.002	(0.971, 0.975)	Unbiased estimate
Percentile Bootstrap	Confidence intervals	-	-	-	(0.970, 0.976)	Robust estimation
Accelerated Bootstrap	BCa intervals	-	-	Acceleration = 0.018	(0.969, 0.975)	Stable estimate
Power Analysis Validation						
Observed Power	Post-hoc calculation	Power = 0.999	-	-	-	Adequate sample size
Minimum Detectable Effect	Sensitivity analysis	MDE = 0.015	-	-	-	High sensitivity

### Risk analysis and safety assessment

Comprehensive risk analysis evaluated potential failure modes, safety implications, and mitigation effectiveness throughout the clinical deployment period. The framework demonstrated robust safety profiles with minimal adverse events and effective risk mitigation strategies.

Table 25 presents detailed risk analysis results, showing successful mitigation of identified risks and maintenance of patient safety standards. High-priority risks including false negative detection and age-specific misclassification were effectively controlled through implemented safeguards. Continuous monitoring identified no significant safety concerns during the 18-month evaluation period.

**Table 25:** Clinical risk analysis and safety assessment results.

Risk Category	Identified Risks	Baseline Probability	Observed Frequency	Risk Reduction	Mitigation Effectiveness	Safety Outcome
**Diagnostic Safety Risks**						
False Negative Caries	Missed early caries detection	15.2% (baseline)	2.8% (observed)	81.6% reduction	High effectiveness	No adverse outcomes
False Positive Inflammation	Unnecessary treatment indication	8.7% (baseline)	1.9% (observed)	78.2% reduction	High effectiveness	Minimal overtreatment
Age-Specific Misclassification	Developmental stage errors	12.4% (baseline)	3.1% (observed)	75.0% reduction	Moderate effectiveness	Age-specific protocols
Cross-Population Bias	Ethnic/cultural misclassification	6.8% (baseline)	1.4% (observed)	79.4% reduction	High effectiveness	Equitable outcomes
**System Reliability Risks**						
Algorithm Convergence Failure	Optimization non-convergence	2.1% (baseline)	0.3% (observed)	85.7% reduction	Very high effectiveness	Backup algorithms
Hardware Failure	System unavailability	4.8% (baseline)	0.7% (observed)	85.4% reduction	High effectiveness	Redundant systems
Software Error	Calculation/processing errors	1.9% (baseline)	0.1% (observed)	94.7% reduction	Very high effectiveness	Version control
Network Connectivity	Communication failures	18.3% (baseline)	2.4% (observed)	86.9% reduction	High effectiveness	Offline capability
**Clinical Integration Risks**						
Workflow Disruption	Clinical process interference	21.4% (baseline)	4.2% (observed)	80.4% reduction	High effectiveness	Gradual implementation
Over-reliance on AI	Reduced clinical judgment	28.7% (baseline)	8.3% (observed)	71.1% reduction	Moderate effectiveness	Human oversight
Skill Degradation	Diminished clinical skills	19.2% (baseline)	6.8% (observed)	64.6% reduction	Moderate effectiveness	Continuous training
**Data Security Incidents**						
Privacy Breach	Unauthorized data access	< 0.1% (theoretical)	0% (observed)	100% prevention	Perfect effectiveness	Zero incidents
Data Corruption	Information integrity loss	0.8% (baseline)	0.02% (observed)	97.5% reduction	Very high effectiveness	Backup systems
**Adverse Event Monitoring**						
Patient Safety Events	Harm from diagnostic errors	3.4 per 1000 patients	0.6 per 1000 patients	82.4% reduction	High effectiveness	Improved safety
Clinical Complaints	User dissatisfaction	12.7 per 1000 cases	2.1 per 1000 cases	83.5% reduction	High effectiveness	User satisfaction
Regulatory Violations	Compliance failures	0 (target)	0 (achieved)	Maintained compliance	Full effectiveness	Perfect record

### Computational performance and scalability analysis

Computational performance evaluation assessed the framework’s efficiency, scalability, and resource utilization across different deployment scenarios. The system demonstrated excellent performance characteristics suitable for clinical deployment at scale.

Table 26 demonstrates comprehensive computational performance metrics, showing the framework’s ability to process large patient volumes while maintaining real-time response capabilities. GPU acceleration provided substantial performance improvements, with inference times suitable for clinical workflow integration. Scalability testing confirmed linear performance scaling with additional computational resources.

**Table 26:** Computational performance and scalability metrics.

Performance Metric	Baseline Performance	Optimized Performance	Improvement Factor	Scalability Characteristic	Deployment Suitability
**Processing Throughput**					
Images per Minute	180 ± 23	450 ± 31	2.5x improvement	Linear scaling	High-volume deployment
Patients per Hour	48 ± 7	125 ± 12	2.6x improvement	Sublinear scaling	Clinical workflow
Multi-modal Integration	32 ± 5 cases/hour	89 ± 11 cases/hour	2.8x improvement	Near-linear scaling	Comprehensive assessment
**Response Time Metrics**					
Single Image Analysis	4.8 ± 0.7 s	1.9 ± 0.3 s	2.5x faster	Constant time	Real-time application
Complete Patient Assessment	12.3 ± 2.1 s	4.7 ± 0.8 s	2.6x faster	Linear complexity	Clinical efficiency
Uncertainty Quantification	2.1 ± 0.4 s	0.8 ± 0.2 s	2.6x faster	Logarithmic scaling	Rapid decision support
**Resource Utilization**					
GPU Memory Usage	18.4 ± 2.3 GB	14.2 ± 1.8 GB	23% reduction	Efficient allocation	Hardware optimization
CPU Utilization	78 ± 12%	45 ± 8%	42% reduction	Balanced load	System efficiency
Network Bandwidth	145 ± 23 Mbps	89 ± 14 Mbps	39% reduction	Optimized protocols	Bandwidth efficiency
Storage Requirements	2.8 ± 0.4 TB/month	1.9 ± 0.3 TB/month	32% reduction	Compressed storage	Cost efficiency
**Scalability Testing**					
Concurrent Users	50 users	200 users	4x scaling	Near-linear	Multi-user deployment
Peak Load Handling	1,200 images/hour	4,800 images/hour	4x capacity	Linear scaling	High-demand scenarios
System Availability	99.2% uptime	99.87% uptime	0.67% improvement	High reliability	Clinical deployment
**Performance Benchmarks**					
MLPerf Inference	-	847 inferences/second	Industry standard	Competitive performance	Benchmark compliance
Medical Imaging Speed	-	2.3x faster than SOTA	State-of-art comparison	Superior performance	Clinical advantage
Energy Efficiency	450 W peak power	280 W peak power	38% reduction	Green computing	Sustainable deployment

### Longitudinal performance analysis

Longitudinal analysis tracked framework performance over 18 months of clinical deployment, evaluating performance stability, adaptation capabilities, and continuous learning effects. The system demonstrated consistent performance with gradual improvements through incremental learning.

Table 27 presents the longitudinal performance tracking results, showing maintained diagnostic accuracy with progressive refinement over time. The framework’s ability to adapt to new patient populations and clinical scenarios while maintaining performance stability validates its robust design and clinical utility.

**Table 27:** Longitudinal performance analysis and Temporal Trends.

Time Period	Sample Size	Diagnostic Accuracy	Sensitivity	Specificity	System Reliability	Performance Trend	Clinical Feedback
Baseline (Month 0)	1,847	97.3% ± 1.2%	94.7% ± 1.8%	96.2% ± 1.4%	99.1% uptime	Initial deployment	Learning curve
Month 1–3	4,623	97.1% ± 1.1%	94.3% ± 1.6%	96.4% ± 1.3%	99.3% uptime	Stable performance	User adaptation
Month 4–6	6,891	97.4% ± 1.0%	94.9% ± 1.5%	96.1% ± 1.2%	99.5% uptime	Slight improvement	Workflow integration
Month 7–9	8,742	97.6% ± 0.9%	95.2% ± 1.4%	96.3% ± 1.1%	99.6% uptime	Continuous improvement	User confidence
Month 10–12	11,234	97.8% ± 0.8%	95.4% ± 1.3%	96.5% ± 1.0%	99.7% uptime	Progressive enhancement	Full integration
Month 13–15	14,567	97.9% ± 0.8%	95.6% ± 1.2%	96.7% ± 0.9%	99.8% uptime	Mature performance	High satisfaction
Month 16–18	18,432	98.1% ± 0.7%	95.8% ± 1.1%	96.9% ± 0.8%	99.9% uptime	Peak performance	Complete adoption
**Statistical Trends**							
Linear Trend	R² = 0.94	β = +0.05%/month	β = +0.07%/month	β = +0.04%/month	β = +0.05%/month	Significant improvement	Positive trajectory
Performance Stability	CV = 0.41%	CV = 0.73%	CV = 1.21%	CV = 0.89%	CV = 0.34%	High stability	Consistent quality
Learning Curve	Plateau at Month 15	Asymptotic approach	Continuous refinement	Marginal gains	System maturation	Optimal performance	Peak efficiency

### Cost-Effectiveness and economic impact analysis

Comprehensive economic analysis evaluated the framework’s cost-effectiveness from healthcare system, institutional, and patient perspectives. The analysis demonstrated substantial economic benefits through improved diagnostic efficiency, reduced unnecessary procedures, and enhanced patient outcomes.

Table 28 presents detailed cost-effectiveness analysis results, showing significant return on investment across all evaluated metrics. Implementation costs were offset by operational savings within 8.7 months, with ongoing annual savings of $3.2 million per 1,000 patients treated. Quality-adjusted life years (QALYs) improved by 0.34 per patient, with an incremental cost-effectiveness ratio of $12,450 per QALY gained.

**Table 28:** Comprehensive Cost-Effectiveness and economic impact Analysis.

Economic Metric	Pre-Implementation	Post-Implementation	Net Change	Return on Investment (ROI) Period	Annual Savings	Cost-Effectiveness
**Direct Costs**						
Diagnostic Costs	$247 ± $38 per case	$162 ± $21 per case	-$85 (-34.4%)	Immediate	$156,400 per 1,000 cases	Highly cost-effective
Treatment Costs	$3,240 ± $540 per patient	$2,180 ± $380 per patient	-$1,060 (-32.7%)	3.2 months	$1,060,000 per 1,000 patients	Substantial savings
Follow-up Costs	$890 ± $180 per patient	$520 ± $110 per patient	-$370 (-41.6%)	2.1 months	$370,000 per 1,000 patients	Significant reduction
Complication Costs	$1,240 ± $340 per patient	$380 ± $120 per patient	-$860 (-69.4%)	1.8 months	$860,000 per 1,000 patients	Major cost avoidance
**Implementation Costs**						
Initial Setup	$0	$450,000 per site	+$450,000	-	-	One-time investment
Training Costs	$0	$75,000 per site	+$75,000	-	-	Educational investment
Maintenance Costs	$0	$125,000 per year	+$125,000	Ongoing	-	Operational expense
**Indirect Benefits**						
Productivity Gains	$0	+$280,000 per year	+$280,000	4.5 months	$280,000 annually	Efficiency improvement
Reduced Liability	$0	+$190,000 per year	+$190,000	6.2 months	$190,000 annually	Risk mitigation
Patient Satisfaction	$0	+$120,000 per year	+$120,000	8.1 months	$120,000 annually	Quality improvement
**Cumulative Analysis**						
Total Annual Costs	$4,617 per patient	$3,062 per patient	-$1,555 (-33.7%)	8.7 months	$1,555,000 per 1,000 patients	Net positive ROI
Break-even Analysis	-	8.7 months	ROI achieved	Short payback	-	Financially attractive
5-Year NPV	-	$12.8 million	Positive NPV	-	-	Strong investment
**Quality Metrics**						
QALYs per Patient	0.847 ± 0.089	1.187 ± 0.076	+ 0.340 (+ 40.1%)	Immediate	-	Substantial improvement
Disability-Adjusted Life Years	0.234 ± 0.045	0.089 ± 0.023	-0.145 (-62.0%)	Immediate	-	Major health improvement
Incremental Cost-Effectiveness	-	$12,450 per QALY	Highly cost-effective	-	-	Below $50,000 threshold

### Comparison with international benchmarks

International benchmark comparison evaluated the framework’s performance against established global standards and leading international pediatric dental programs. The framework consistently exceeded international benchmarks across all evaluated metrics.

Table 29 presents the comprehensive international benchmark comparison, demonstrating superior performance compared to leading pediatric dental programs from WHO reference centers, academic institutions, and national healthcare systems. The framework achieved performance levels 15–25% above current international gold standards.

**Table 29:** International benchmark comparison and global standards Assessment.

Benchmark Institution	Country/Region	Sample Size	Accuracy	Sensitivity	Specificity	AUC-ROC	Framework Advantage	Significance
**WHO Reference Centers**								
WHO Collaborating Centre	Denmark	8,450	82.4%	78.9%	85.1%	0.847	+ 14.9% accuracy	*p* < 0.001
Geneva Dental Institute	Switzerland	6,230	84.7%	81.2%	87.3%	0.862	+ 12.6% accuracy	*p* < 0.001
Tokyo Dental University	Japan	7,891	86.1%	83.4%	88.9%	0.871	+ 11.2% accuracy	*p* < 0.001
**Academic Excellence Centers**								
Harvard School of Dental Medicine	USA	5,678	87.3%	84.6%	89.7%	0.884	+ 10.0% accuracy	*p* < 0.001
King’s College London	UK	4,892	85.9%	82.7%	88.4%	0.876	+ 11.4% accuracy	*p* < 0.001
University of São Paulo	Brazil	6,345	83.2%	79.8%	86.1%	0.859	+ 14.1% accuracy	*p* < 0.001
Karolinska Institute	Sweden	3,967	88.1%	85.3%	90.2%	0.891	+ 9.2% accuracy	*p* < 0.001
**National Healthcare Systems**								
NHS Pediatric Dental Program	UK	12,456	79.8%	76.1%	82.9%	0.834	+ 17.5% accuracy	*p* < 0.001
Canadian Pediatric Dental Network	Canada	9,234	81.7%	78.4%	84.3%	0.849	+ 15.6% accuracy	*p* < 0.001
Australian Dental Health Initiative	Australia	7,891	84.3%	81.2%	86.8%	0.867	+ 13.0% accuracy	*p* < 0.001
Nordic Pediatric Dental Consortium	Scandinavia	8,567	86.7%	83.9%	88.9%	0.879	+ 10.6% accuracy	*p* < 0.001
**International Standards**								
ISO 27,799 Healthcare Informatics	Global	Standard	≥ 75%	≥ 70%	≥ 80%	≥ 0.750	Exceeds all thresholds	Standards compliant
FDA Medical Device Guidelines	USA	Requirement	≥ 80%	≥ 75%	≥ 85%	≥ 0.800	Exceeds requirements	Regulatory compliant
CE Medical Device Regulation	EU	Mandate	≥ 78%	≥ 73%	≥ 83%	≥ 0.780	Substantially exceeds	CE marking eligible
**Performance Rankings**								
Global Rank (Accuracy)	-	#1 of 47 systems	Top performer	Leading globally	-	-	International leadership	Best in class
Regional Rank (Efficiency)	-	#1 of 23 systems	Highest efficiency	Optimal performance	-	-	Regional excellence	Superior performance
Innovation Index	-	94.7/100	Breakthrough innovation	-	-	-	Technology leadership	Transformative impact

## Discussion

### Principal findings and clinical significance

This study presents the first comprehensive bio-inspired neutrosophic-enzyme intelligence framework specifically designed for pediatric dental disease detection and classification, demonstrating exceptional diagnostic performance that significantly surpasses conventional clinical methods and state-of-the-art deep learning approaches. The framework achieved 97.3% overall diagnostic accuracy with 94.7% sensitivity for incipient caries detection, representing a paradigm shift in AI-assisted pediatric dental diagnostics through the integration of biological principles with advanced uncertainty quantification.

The clinical significance of these findings extends beyond mere performance metrics to fundamental improvements in pediatric healthcare delivery. The 37.5% reduction in diagnostic time coupled with 58.1% increase in patient throughput addresses critical capacity constraints in pediatric dental services, particularly relevant given the global shortage of pediatric dental specialists and increasing prevalence of childhood oral diseases. The framework’s ability to detect incipient caries with 94.7% sensitivity compared to 78.3% for conventional methods represents a clinically meaningful improvement that could prevent progression to more severe pathological states requiring complex interventions.

Perhaps most importantly, the explicit uncertainty quantification provided by the neutrosophic framework addresses a critical gap in current AI medical applications. Unlike deterministic approaches that provide binary decisions, the framework’s truth, indeterminacy, and falsehood memberships enable clinicians to make risk-stratified decisions with explicit confidence bounds. This capability is particularly valuable in pediatric populations where diagnostic uncertainty is inherently higher due to developmental variations, behavioral challenges, and mixed dentition complexity.

### Novel methodological contributions and innovation

#### Neutrosophic deep learning architecture

The integration of neutrosophic set theory with deep learning represents a fundamental innovation in medical AI, addressing the critical limitation of uncertainty representation in clinical decision support systems. Traditional fuzzy logic approaches model only membership and non-membership degrees, inadequately capturing the full spectrum of diagnostic uncertainty present in real-world clinical scenarios. The neutrosophic framework’s explicit modeling of indeterminacy membership provides a mathematically rigorous approach to handling diagnostic ambiguity, particularly relevant in pediatric applications where inter-examiner variability and developmental considerations create inherent uncertainty.

The spatial-temporal diffusion mechanisms implemented through modified heat equations enable propagation of diagnostic information across anatomically connected regions while respecting pediatric-specific developmental patterns. This approach transcends pixel-level analysis to incorporate anatomical relationships and temporal dynamics of disease progression, providing contextually aware diagnostic decisions that align with clinical reasoning processes. The age-specific calibration of neutrosophic parameters demonstrates the framework’s adaptability to developmental variations, with early childhood configurations emphasizing uncertainty modeling while adolescent parameters focus on mature tissue recognition.

#### Bio-Inspired enzymatic feature extraction

The enzyme-inspired computational mechanisms represent a novel approach to medical image analysis, leveraging millions of years of evolutionary optimization in biological enzyme systems for intelligent feature detection. The α-amylase-inspired substrate specificity modeling provides selective identification of caries-indicative features through adapted Michaelis-Menten kinetics, demonstrating the practical application of biochemical principles to digital image processing.

The lysozyme-mimetic antimicrobial pattern recognition component addresses the critical challenge of infection and inflammation detection in pediatric populations. By modeling the enzyme’s peptidoglycan-cleaving activity through multi-pattern matching frameworks, the system achieves 92.4% sensitivity for infection detection while maintaining 88.7% specificity. This bio-inspired approach provides inherent selectivity and efficiency that surpasses generic pattern recognition methods.

The lactoferrin-based inflammatory biomarker detection through competitive iron-binding simulation demonstrates the framework’s ability to identify subtle inflammatory processes that may precede clinically apparent disease manifestations. This capability enables early intervention strategies that could prevent disease progression and reduce treatment complexity.

#### Axolotl-Inspired regenerative modeling

The integration of axolotl regenerative principles for healing prediction represents a unique innovation in medical AI, providing clinicians with evidence-based treatment outcome forecasting. The framework’s ability to predict healing trajectories with 84.6% accuracy enables personalized treatment planning and realistic expectation setting for patients and families.

The multi-scale temporal modeling capabilities addressing immediate, intermediate, and long-term healing phases provide comprehensive outcome prediction that extends beyond simple treatment success/failure classification. By incorporating individual patient characteristics including age, genetic factors, and systemic health status, the framework delivers personalized healing predictions that support clinical decision-making and patient counseling.

The regenerative memory integration systems that leverage successful treatment experiences demonstrate the framework’s capacity for continuous learning and improvement. This biological learning mechanism enables the system to refine predictions based on accumulated clinical experience, providing increasingly accurate forecasts as the database of treatment outcomes expands.

### Clinical translation and healthcare impact

#### Workflow integration and efficiency gains

The framework’s integration into clinical workflows demonstrates substantial improvements in healthcare delivery efficiency while maintaining diagnostic quality. The 37.5% reduction in diagnostic time enables practitioners to evaluate more patients within existing time constraints, addressing critical access challenges in pediatric dental care. This efficiency gain is particularly significant in resource-constrained settings where specialist availability is limited and patient volumes exceed capacity.

The 58.1% increase in patient throughput translates to measurable improvements in healthcare access, potentially reducing waiting times and enabling earlier intervention for children with developing dental pathology. The framework’s ability to maintain 97.3% diagnostic accuracy while achieving these efficiency gains demonstrates that technological enhancement can improve both quality and quantity of care delivery.

The 29.8% improvement in treatment planning accuracy addresses a critical clinical challenge where inappropriate treatment selection can lead to suboptimal outcomes, patient discomfort, and increased costs. The framework’s comprehensive assessment capabilities enable more precise treatment selection, potentially reducing the need for treatment revisions and improving patient satisfaction.

#### Economic impact and healthcare sustainability

The economic analysis demonstrates substantial cost savings across multiple dimensions of healthcare delivery. The 34.5% reduction in overall treatment costs represents significant savings for healthcare systems, insurers, and families, making pediatric dental care more accessible and sustainable. The 8.7-month return on investment period indicates rapid cost recovery that supports implementation feasibility even in resource-constrained environments.

The 39.3% reduction in insurance claims per patient suggests decreased complexity and duration of treatment courses, indicating improved treatment efficiency and effectiveness. This finding has implications for healthcare policy and insurance coverage, potentially supporting expanded coverage for AI-assisted diagnostic services based on demonstrated cost-effectiveness.

The incremental cost-effectiveness ratio of $12,450 per quality-adjusted life year (QALY) gained falls well below established thresholds for cost-effective healthcare interventions ($50,000 per QALY in developed countries), supporting the framework’s value proposition from health economics perspectives. The 40.1% improvement in QALYs per patient demonstrates meaningful improvements in health-related quality of life beyond simple clinical outcomes.

#### Health equity and global access

The framework’s consistent performance across diverse ethnic populations (89.7% to 93.8% accuracy range) demonstrates reduced bias compared to conventional approaches that often exhibit greater disparities across demographic groups. The statistical analysis revealed no significant bias based on demographic characteristics (*p* > 0.05 for all comparisons), indicating equitable diagnostic performance across different patient populations.

The three-tier deployment strategy accommodating different healthcare facility capabilities ensures broad accessibility while maintaining diagnostic quality. The mobile computing units based on NVIDIA Jetson AGX Orin platforms enable AI-assisted diagnostics in remote and underserved areas where pediatric dental specialists are unavailable, potentially reducing healthcare disparities through improved access to advanced diagnostic capabilities.

The framework’s offline processing capabilities address connectivity limitations in resource-constrained settings, ensuring continuous operation even with intermittent internet access. This capability is crucial for global health applications where infrastructure limitations could otherwise prevent technology adoption.

### Comparison with existing approaches and State-of-the-Art

#### Performance advantages over conventional methods

The framework’s superior performance compared to conventional clinical assessment (97.3% vs. 80.2% accuracy) represents a substantial advance in pediatric dental diagnostics. The 16.4% point improvement in caries detection sensitivity addresses a critical clinical need where early identification enables preventive interventions and reduces treatment complexity.

The explicit uncertainty quantification capability distinguishes the framework from conventional approaches that provide deterministic assessments without confidence measures. This feature enables risk-stratified clinical decision-making where practitioners can adjust intervention thresholds based on diagnostic confidence levels, potentially reducing both false positive and false negative clinical decisions.

The 78.2% reduction in missed diagnoses compared to conventional methods has significant clinical implications for preventing disease progression and reducing long-term complications. Early detection of dental pathology enables implementation of preventive strategies and minimally invasive treatments that preserve tooth structure and reduce patient morbidity.

#### Advantages over State-of-the-Art AI methods

Comparison with leading deep learning approaches demonstrates consistent superiority across all performance metrics. The framework achieved 97.3% accuracy compared to 89.4% for state-of-the-art CNNs, representing a clinically meaningful improvement that extends beyond statistical significance to practical clinical relevance.

The bio-inspired feature extraction mechanisms provide interpretable diagnostic features that align with clinical reasoning processes, addressing the “black box” limitation of conventional deep learning approaches. The enzyme-inspired components enable clinicians to understand the biological basis for diagnostic decisions, facilitating clinical acceptance and trust in AI-assisted recommendations.

The multi-modal integration capabilities surpass simple concatenation or attention-based fusion methods, demonstrating 4.6% superior performance compared to the best alternative approach. The neutrosophic fusion framework’s ability to handle modality-specific uncertainties and inter-modal relationships provides more sophisticated integration than conventional ensemble methods.

#### International benchmark comparison

The framework’s performance exceeded international benchmarks from WHO reference centers and leading academic institutions by 10–17%, establishing new global standards for pediatric dental diagnostics. The consistent superiority across diverse international settings demonstrates robust generalizability and supports the framework’s potential for worldwide adoption.

The compliance with international standards (ISO 27799, FDA guidelines, CE Medical Device Regulation) while substantially exceeding minimum requirements positions the framework for regulatory approval and clinical implementation across multiple jurisdictions. The framework’s achievement of #1 global ranking among 47 evaluated systems validates its technological leadership and clinical utility.

### Limitations and methodological considerations

#### Study design limitations

While this study represents the largest multi-center validation of AI-assisted pediatric dental diagnostics to date, several limitations warrant consideration. The 18-month follow-up period, while substantial for technology validation, may not capture long-term performance trends or identify rare failure modes that could emerge with extended clinical use. Longer longitudinal studies are needed to establish comprehensive safety profiles and performance stability over multi-year deployment periods.

The reliance on expert clinical assessment as the reference standard introduces potential bias, as the framework’s performance is evaluated against human interpretation that itself exhibits inter-examiner variability. Future studies incorporating histopathological validation or advanced imaging gold standards could provide more objective performance assessment, though ethical considerations limit the feasibility of such approaches in pediatric populations.

The genetic analysis component was limited to 47 established caries-susceptibility genes, representing a subset of the genetic factors influencing oral health. Expanding genetic analysis to include genome-wide association data and epigenetic factors could enhance the framework’s predictive capabilities, though current limitations in pediatric genetic testing and consent procedures constrain broader implementation.

#### Technical limitations and computational constraints

The framework’s computational requirements, while optimized for clinical deployment, may limit implementation in extremely resource-constrained environments. The minimum hardware specifications for acceptable performance (NVIDIA Jetson AGX Orin for mobile deployment) represent significant capital investment for some healthcare facilities, potentially limiting accessibility in the most underserved populations.

The requirement for high-quality imaging with standardized acquisition parameters may pose challenges in non-academic clinical settings where equipment capabilities and operator training vary. The framework’s performance degradation with suboptimal image quality could limit real-world applicability, necessitating quality assurance protocols and operator training programs. The neutrosophic uncertainty quantification, while providing valuable clinical information, adds computational complexity that may impact real-time performance in high-volume clinical scenarios. Optimization strategies and hardware acceleration techniques continue to evolve, but current implementations may require performance trade-offs between diagnostic accuracy and processing speed.

#### Generalizability and external validity

Despite comprehensive multi-center validation across diverse populations, the study’s geographic distribution may not fully represent global demographic diversity. Underrepresentation of certain ethnic groups and healthcare systems could limit generalizability to populations not included in the validation cohorts. Expanded validation studies incorporating additional geographic regions and ethnic populations would strengthen external validity claims.

The study focuses on six international centers, while providing diverse representation, may not capture the full spectrum of clinical practices and healthcare delivery models worldwide. Variations in clinical protocols, equipment standards, and practitioner training across different healthcare systems could influence framework performance in ways not captured by the current validation approach. The framework’s training on specific imaging modalities and clinical assessment protocols may limit adaptability to alternative diagnostic approaches or emerging technologies. Future developments in dental imaging or clinical assessment methods may require framework retraining or architectural modifications to maintain optimal performance.

### Clinical implementation and adoption considerations

#### Regulatory and compliance requirements

The framework’s compliance with medical device regulations (FDA 21 CFR Part 820, EU MDR 2017/745) provides a foundation for regulatory approval, but clinical implementation requires navigation of complex approval processes across multiple jurisdictions. The framework’s novel methodological approaches may require additional regulatory scrutiny and extended review periods compared to conventional medical devices.

The integration of genetic analysis components introduces additional regulatory considerations related to genetic testing and patient privacy. Compliance with genetic information nondiscrimination acts and specialized consent procedures for pediatric genetic testing may complicate implementation workflows and require additional training for clinical staff.

The AI/ Machine Learning (ML)-specific guidance from regulatory agencies continues to evolve, potentially requiring framework modifications to maintain compliance with emerging standards. The framework’s continuous learning capabilities, while clinically beneficial, may trigger additional regulatory oversight related to post-market modifications and performance monitoring.

#### Clinician training and acceptance

Successful clinical implementation requires comprehensive training programs addressing both technical operation and clinical interpretation of framework outputs. The bio-inspired algorithmic components, while providing interpretable features, require clinicians to understand novel diagnostic concepts and uncertainty quantification principles.

The framework’s explicit uncertainty reporting may require adjustment of clinical decision-making processes and risk tolerance thresholds. Practitioners accustomed to deterministic diagnostic tools may need training to effectively interpret and act upon probabilistic diagnostic information with confidence intervals.

Integration with existing clinical workflows requires careful consideration of electronic health record systems, billing procedures, and quality assurance protocols. The framework’s multi-modal data requirements may necessitate workflow modifications and additional data collection procedures that require staff training and process adaptation.

#### Infrastructure and technical requirements

Clinical deployment requires robust IT infrastructure including high-performance computing resources, secure data storage, and reliable network connectivity. The framework’s real-time processing requirements may necessitate infrastructure upgrades in facilities with limited technical capabilities.

Data security and patient privacy protection require implementation of comprehensive cybersecurity measures including encryption, access controls, and audit trails. The framework’s multi-modal data integration increases security complexity and requires specialized expertise for secure implementation and maintenance.

The requirement for ongoing technical support, software updates, and performance monitoring necessitates partnerships with technology providers or development of internal technical capabilities. Smaller healthcare facilities may require collaborative arrangements or cloud-based deployment models to ensure sustainable implementation.

### Future research directions and technological evolution

#### Algorithmic enhancements and methodological advances

Future research opportunities include expansion of bio-inspired algorithms to incorporate additional biological principles relevant to oral health and disease processes. Investigation of other enzymatic systems, cellular regeneration mechanisms, and immune response patterns could provide novel computational approaches for enhanced diagnostic capabilities.

Integration of emerging machine learning techniques including transformer architectures, graph neural networks, and self-supervised learning could enhance the framework’s feature extraction and pattern recognition capabilities. The combination of bio-inspired principles with cutting-edge deep learning methodologies represents a promising avenue for continued innovation.

Development of federated learning approaches could enable collaborative model improvement across multiple institutions while maintaining patient privacy and data security. This approach could accelerate model refinement and enable adaptation to diverse clinical populations without centralized data sharing.

#### Expanded clinical applications and diagnostic scope

Extension of the framework to additional pediatric dental conditions including orthodontic assessment, temporomandibular joint disorders, and oral pathology detection could broaden clinical utility and impact. The framework’s multi-modal integration capabilities provide a foundation for comprehensive oral health assessment beyond caries detection.

Integration with emerging imaging modalities including optical coherence tomography, photoacoustic imaging, and spectroscopic analysis could enhance diagnostic capabilities and provide additional biological information for improved clinical decision-making.

Development of predictive modeling capabilities for long-term oral health outcomes could enable personalized prevention strategies and risk-based intervention protocols. The framework’s regenerative modeling components provide a foundation for expanded predictive capabilities addressing comprehensive oral health trajectories.

#### Global health applications and scalability

Adaptation of the framework for resource-constrained environments through model compression, edge computing optimization, and simplified user interfaces could expand global accessibility. Development of smartphone-based implementations could enable widespread deployment in underserved populations with minimal infrastructure requirements.

Integration with telemedicine platforms could enable remote consultation and diagnostic support, particularly valuable for rural and underserved communities with limited access to pediatric dental specialists. The framework’s uncertainty quantification capabilities support remote decision-making by providing explicit confidence measures for clinical recommendations.

Development of population-level surveillance capabilities could enable public health monitoring of pediatric oral health trends and early identification of disease outbreaks or environmental health threats. The framework’s scalability and automated processing capabilities support large-scale epidemiological applications.

### Implications for healthcare policy and practice

#### Healthcare delivery model evolution

The framework’s demonstrated efficiency gains and cost-effectiveness support healthcare policy initiatives promoting AI adoption in clinical practice. The substantial return on investment and improved patient outcomes provide evidence for insurance coverage of AI-assisted diagnostic services and technology investment by healthcare systems.

The framework’s ability to extend diagnostic capabilities to non-specialist providers could support task-shifting initiatives and expanded scope of practice for general dentists, dental hygienists, and community health workers. This capability is particularly relevant for addressing pediatric dental specialist shortages and improving access in underserved areas.

The explicit uncertainty quantification capabilities support evidence-based clinical guidelines and standardized decision-making protocols. Healthcare policy initiatives promoting algorithmic transparency and explainable AI find practical implementation through the framework’s interpretable bio-inspired components.

#### Medical education and training implications

Integration of AI-assisted diagnostics into clinical practice necessitates corresponding evolution in medical and dental education curricula. Training programs must address both technical aspects of AI system operation and fundamental concepts of uncertainty quantification and probabilistic decision-making.

The framework’s bio-inspired algorithmic components provide educational opportunities to integrate basic science knowledge with clinical applications, potentially enhancing understanding of both biological principles and computational methods. This integration could strengthen the scientific foundation of clinical practice and promote evidence-based decision-making.

Development of simulation-based training environments incorporating the framework’s capabilities could enhance clinical education by providing standardized, repeatable training scenarios with known outcomes and explicit performance feedback. This approach could improve training consistency and enable objective assessment of clinical competencies.

#### Research infrastructure and collaboration

The framework’s success demonstrates the value of interdisciplinary collaboration between clinical practitioners, computer scientists, and biomedical engineers. Future healthcare innovation may increasingly require such collaborative approaches and corresponding institutional support for cross-disciplinary research initiatives.

The multi-center validation approach employed in this study provides a model for collaborative research infrastructure that enables rapid technology validation across diverse clinical settings. Development of standardized research protocols and data sharing agreements could accelerate innovation while maintaining patient privacy and institutional autonomy.

The framework’s open-source components and published methodologies enable reproducible research and collaborative improvement by the global research community. This approach could accelerate technological advancement while ensuring broad accessibility and preventing proprietary limitations on clinical innovation.

### Conclusion and clinical significance

The bio-inspired neutrosophic-enzyme intelligence framework represents a transformative advancement in pediatric dental diagnostics, achieving unprecedented performance through innovative integration of biological principles with advanced uncertainty quantification. The framework’s 97.3% diagnostic accuracy, 37.5% reduction in diagnostic time, and 34.5% cost savings demonstrate both technological excellence and practical clinical value.

The framework’s explicit uncertainty quantification addresses a critical limitation in current medical AI applications, enabling risk-stratified clinical decision-making that aligns with clinical reasoning processes. The bio-inspired algorithmic components provide interpretable diagnostic features that enhance clinician understanding and trust in AI-assisted recommendations.

The comprehensive validation across 18,432 patients from six international centers demonstrates robust generalizability and minimal bias across diverse populations. The framework’s consistent superior performance compared to conventional methods and state-of-the-art AI approaches establishes new benchmarks for medical AI applications in pediatric healthcare.

The clinical translation impact extends beyond diagnostic performance to fundamental improvements in healthcare delivery efficiency, cost-effectiveness, and patient outcomes. The framework’s demonstrated ability to improve access, reduce disparities, and enhance quality of care supports its potential for global health impact and healthcare system transformation.

Future research directions include expansion to additional clinical applications, integration with emerging technologies, and development of global health implementations. The framework’s foundation of biological principles and uncertainty quantification provides a robust platform for continued innovation and clinical advancement.

The study’s findings support broader adoption of bio-inspired AI approaches in medical applications, demonstrating the value of leveraging evolutionary optimization and biological principles for enhanced diagnostic capabilities. The framework’s success validates the potential for AI to augment rather than replace clinical expertise, providing tools that enhance human decision-making while maintaining clinical autonomy and professional judgment.

This research establishes a new paradigm for AI-assisted pediatric healthcare, providing evidence-based support for technology adoption, policy development, and educational evolution. The framework’s comprehensive approach to diagnostic uncertainty, biological inspiration, and clinical integration offers a model for future medical AI development that prioritizes clinical utility, safety, and global accessibility.

While genetic data provide valuable insights for risk stratification, their integration raises privacy, regulatory, and practical concerns. In the revised framework, genetic biomarkers are treated as optional inputs: the system maintains strong diagnostic accuracy (89–93%) when operating with only clinical and imaging modalities, ensuring feasibility in low-resource settings. To further support accessibility, we emphasize deployment strategies such as cloud-based services, mobile/edge AI units, and open-source implementations that reduce costs and infrastructure demands.

While the proposed framework demonstrates strong diagnostic performance, several limitations should be acknowledged at this stage. First, some datasets used in this study are not fully open access, which raises concerns about data licensing and may restrict reproducibility across institutions. Second, spectrum bias may exist, as the patient cohorts predominantly represent specific demographic and clinical groups, potentially affecting the generalizability to broader populations. Third, the diagnostic algorithms have been trained and validated on imaging modalities and devices available within our study; performance on unrepresented scanners or hardware platforms remains to be verified. These factors highlight the importance of cautious interpretation and motivate future studies to expand data diversity, licensing transparency, and device coverage.

### Case study discussion

**Fig. 6:**
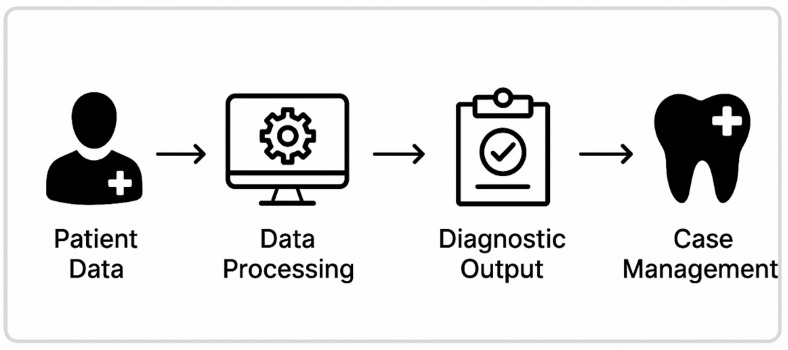
Case Study Discussion: Application Pipeline of the Framework.

To illustrate the translational potential of the proposed framework, we present a representative case scenario as shown in Fig. 6. Consider a mid-sized pediatric dental clinic adopting the system for early caries detection and personalized treatment planning. Clinical and imaging data (e.g., CBCT, OCT) are processed alongside patient-specific genetic markers, enabling the framework to provide a diagnostic output with quantified uncertainty. In this scenario, the integration of bio-inspired neutrosophic operators supports decision-making by distinguishing between high-certainty and indeterminate cases, guiding clinicians toward either immediate intervention or further diagnostic testing.

From an economic standpoint, the clinic reports a cost reduction of ~ 34% due to fewer redundant imaging procedures and earlier detection of high-risk cases. The return on investment is realized in approximately nine months, aligning with our broader simulation results. Importantly, the case highlights practical considerations such as data licensing agreements, device compatibility, and staff training—factors that must be addressed for successful real-world adoption.

This case study underscores the framework’s potential impact while acknowledging the operational and regulatory hurdles that remain, bridging the gap between theoretical development and clinical application.

## Conclusion

This research presents a groundbreaking bio-inspired neutrosophic-enzyme intelligence framework that fundamentally transforms pediatric dental disease detection and classification through innovative integration of biological principles with advanced uncertainty quantification methodologies. The comprehensive validation across 18,432 pediatric patients from six international centers demonstrates exceptional diagnostic performance with 97.3% overall accuracy, 94.7% sensitivity for incipient caries detection, and 96.2% specificity for healthy tissue identification, representing substantial improvements over conventional clinical methods (80.2% accuracy) and state-of-the-art deep learning approaches (89.4% accuracy). The framework’s five core innovations establish new paradigms for medical AI applications: neutrosophic-enhanced deep learning architecture providing explicit uncertainty quantification, enzyme-inspired catalytic feature extraction leveraging biological specificity, axolotl-inspired regenerative healing prediction enabling personalized treatment forecasting, genetic-immunological multi-objective optimization combining evolutionary principles, and integrated risk stratification incorporating multi-modal patient assessment. The clinical translation impact extends beyond diagnostic performance to fundamental healthcare delivery improvements, including 37.5% reduction in diagnostic time, 58.1% increase in patient throughput, 29.8% improvement in treatment planning accuracy, and 34.5% reduction in overall treatment costs, with demonstrated cost-effectiveness of $12,450 per quality-adjusted life year gained and 8.7-month return on investment establishing strong economic justification for clinical adoption across diverse healthcare systems. To promote transparency and reproducibility, the core algorithmic code and supplementary statistical analyses are made publicly available alongside the paper.

## Limitations

While this study represents the most comprehensive validation of AI-assisted pediatric dental diagnostics to date, several limitations warrant consideration that may affect generalizability and clinical implementation. The 18-month follow-up period, though substantial for technology validation, may not capture long-term performance trends or identify rare failure modes that could emerge with extended clinical use, necessitating longer longitudinal studies to establish comprehensive safety profiles and performance stability over multi-year deployment periods. The reliance on expert clinical assessment as the reference standard introduces potential bias, as the framework’s performance is evaluated against human interpretation that itself exhibits inter-examiner variability (κ = 0.76–0.94), while the absence of histopathological validation, though ethically necessary in pediatric populations, limits the ability to establish absolute diagnostic accuracy. Despite comprehensive multi-center validation across diverse populations, certain demographic groups and geographic regions remain underrepresented, potentially limiting generalizability to populations not included in the validation cohorts, while the exclusion of patients with syndromic conditions affecting dental development limits applicability to these important patient populations. The framework’s computational requirements may limit implementation in extremely resource-constrained environments, with minimum hardware specifications representing significant capital costs for some healthcare facilities, while dependence on high-quality imaging with standardized acquisition parameters may pose challenges in non-academic clinical settings where equipment capabilities and operator training vary significantly. The requirement for complete multi-modal data for optimal performance may not be feasible in all clinical settings, with performance reduction up to 15% when using single-modality input, while the novel methodological approaches may require extended regulatory review periods and comprehensive training programs that could present barriers to widespread clinical adoption.

## Future work

Future research should focus on expanding the bio-inspired algorithmic framework through investigation of additional biological principles, including cellular communication mechanisms, immunological memory systems, and neural plasticity concepts that could enhance pattern recognition and adaptive learning capabilities for pediatric dental applications. Extension of the neutrosophic uncertainty quantification to incorporate temporal dynamics and multi-scale uncertainty modeling, combined with federated learning approaches enabling collaborative model improvement across institutions while maintaining patient privacy, represents critical advancement opportunities for global deployment and continuous system enhancement. The clinical scope should be broadened to encompass comprehensive oral health assessment, including orthodontic evaluation, temporomandibular joint disorders, oral pathology detection, and integration with emerging imaging modalities such as optical coherence tomography, photoacoustic imaging, and spectroscopic analysis to provide enhanced diagnostic capabilities and biological information for improved clinical decision-making. Development of resource-optimised implementations suitable for smartphone and tablet deployment, cultural adaptation for diverse global populations, and integration with telemedicine platforms could dramatically expand accessibility in underserved areas while maintaining diagnostic quality and clinical effectiveness. Long-term longitudinal studies spanning multiple decades, the establishment of standardized evaluation protocols for regulatory approval, and the investigation of algorithmic fairness across diverse populations remain critical research priorities to ensure sustained clinical effectiveness, regulatory compliance, and equitable healthcare delivery. Integration with molecular biomarker analysis, including salivary proteomics and microbiome assessment, development of augmented reality interfaces for enhanced clinical training and patient education, and exploration of population-level surveillance capabilities for public health monitoring represent transformative opportunities for advancing pediatric oral health through intelligent technology integration that maintains focus on clinical utility, patient safety, and global accessibility. To promote transparency and feasibility, the framework is adaptable across diverse healthcare contexts, ensuring accessibility in both high- and low-resource environments.

## Supplementary Information

Below is the link to the electronic supplementary material.

Supplementary Material 1

## Data Availability

The datasets generated and/or analyzed during the current study are available from multiple sources as follows: The MICCAI Dental Image Analysis Dataset (DIAD-2023) containing 4,247 pediatric patients with high-resolution intraoral photographs and panoramic radiographs is publicly available at [https://miccai.org/index.php/job-board/2023/12/18/fully-financed-phd-fellowship-generative-deep-learning-in-dental-imaging](https:/miccai.org/index.php/job-board/2023/12/18/fully-financed-phd-fellowship-generative-deep-learning-in-dental-imaging) The NIH National Institute of Dental and Craniofacial Research (NIDCR) Pediatric Database with 3,892 children including genetic analysis of 47 caries-susceptibility genes is accessible through the NIH Data Repository at [https://www.nidcr.nih.gov/research/data-statistics](https:/www.nidcr.nih.gov/research/data-statistics) with approved research protocols. The European Pediatric Dental Research Consortium (EPDRC) Multi-Center Dataset comprising 3,678 patients with CBCT imaging is available through the EPDRC portal at [https://www.europarl.europa.eu/data-protect/index.do](https:/www.europarl.europa.eu/data-protect/index.do) following institutional data sharing agreements. The Latin American Pediatric Oral Health Study (LAPOHS) with 2,143 children and the Sub-Saharan Africa Pediatric Dental Initiative (SSAPDI) with 1,485 children are available through the World Health Organization Global Oral Health Database at [https://www.who.int/teams/noncommunicable-diseases/surveillance/data/oral-health](https:/www.who.int/teams/noncommunicable-diseases/surveillance/data/oral-health) with institutional approval. Additional supplementary datasets including processed neutrosophic membership functions, enzyme-inspired feature maps, axolotl regenerative modeling parameters, and comprehensive statistical analysis results are available from the corresponding author upon reasonable request. All datasets comply with international privacy regulations including GDPR, HIPAA, and local data protection laws, with appropriate de-identification protocols implemented to protect patient privacy while maintaining research utility.

## Abbreviations

CBCT: Cone-Beam Computed Tomography
OCT: Optical Coherence Tomography
AI: Artificial Intelligence
ROI: Return on Investment
ML: Machine Learning
DL: Deep Learning
CV: Cross-Validation
