# Supplementary material for: Bio-inspired neutrosophic-enzyme intelligence framework for pediatric dental disease detection using multi-modal clinical data
Source: Sci Rep. 2025 Oct 17;15:36299. doi: 10.1038/s41598-025-21923-5 (PMC12534394; doi:10.1038/s41598-025-21923-5)
Supplement: Supplementary file 1 — Supplementary Material 1 [file 41598_2025_21923_MOESM1_ESM.docx]

**Algorithm 1:** Bio-Inspired Neutrosophic-Enzyme Pediatric Dental Diagnostic Framework

1: Initialize neutrosophic membership functions T, I, F for all patients

2: Initialize enzyme-inspired feature extraction parameters [α, λ, φ]

3: Initialize axolotl regenerative potential field R₀ for healing prediction

4: Initialize genetic-immunological optimization population

5: Set multi-objective diagnostic weights $\omega= \left[ \omega_{\mathrm{accuracy}}, \omega_{\mathrm{uncertainty}}, \omega_{\mathrm{clinical}} \right]$

6: t ← 0

7: repeat

8: // Phase 1: Multi-Modal Data Preprocessing

9: Normalize clinical data X_clinical using age-specific ranges

10: Enhance radiographic images$X_{\mathrm{radio}}$ with pediatric-specific filters

11: Process genetic markers X_genetic through inheritance modeling

12: Standardize behavioral data $X_{\mathrm{behavioral}}$ across developmental stages

13:

14: // Phase 2: Neutrosophic Uncertainty Modeling

15: Update truth membership T based on diagnostic confidence

16: Update indeterminacy membership I for uncertain pathological regions

17: Update falsehood membership F for healthy tissue identification

18: Apply spatial-temporal diffusion to neutrosophic diagnostic fields

19:

20: // Phase 3: Enzyme-Inspired Feature Extraction

21: for each patient p_i do

22: // α-Amylase-inspired caries detection

23: $F_{\mathrm{caries}}\leftarrow\mathrm{substrate}_{\mathrm{specificity}_{\mathrm{model}\left( X_{\mathrm{radio}\left[ i \right]}, \alpha_{\mathrm{amylase}_{\mathrm{params}}} \right)}}$

24: // Lysozyme-mimetic infection assessment

25: $F_{\mathrm{infection}}\leftarrow\mathrm{antimicrobial}_{\mathrm{pattern}_{\mathrm{recognition}\left( X_{\mathrm{clinical}\left[ i \right]}, \mathrm{lysozyme}_{\mathrm{params}} \right)}}$

26: // Lactoferrin-based inflammation detection

27: $F_{\mathrm{inflammation}}\leftarrow\mathrm{iron}_{\mathrm{binding}_{\mathrm{simulation}\left( X_{\mathrm{genetic}\left[ i \right]}, \mathrm{lactoferrin}_{\mathrm{params}} \right)}}$

28: // Catalytic efficiency optimization

29: Optimize enzyme parameters based on local tissue characteristics

30: end for

31:

32: // Phase 4: Axolotl-Inspired Healing Prediction

33: for each treatment scenario s_j do

34: Compute regenerative potential R(t, age_i, genetics_i)

35: Apply temporal adaptation for healing trajectory prediction

36: Model tissue regeneration using axolotl-inspired mechanisms

37: Integrate individual healing capacity factors

38: end for

39:

40: // Phase 5: Genetic-Immunological Optimization

41: Update territorial boundaries based on diagnostic performance

42: Resolve feature selection conflicts through immune hierarchy

43: Select optimal biomarkers within genetic constraints

44: Update population fitness based on clinical validation feedback

45:

46: // Phase 6: Risk Stratification and Personalization

47: Integrate family history through multigenerational modeling

48: Assess environmental factors and behavioral patterns

49: Compute personalized risk scores R_risk = f(genetics, environment, behavior)

50: Optimize treatment protocols based on individual characteristics

51:

52: // Phase 7: Integrated Decision Support and Validation

53: Compute hybrid diagnostic probabilities with uncertainty bounds

54: Update dynamic component weights ω(t+1) based on performance

55: Integrate solutions from all framework components

56: Generate clinical decision support recommendations

57:

58: // Quality Assessment and Convergence Check

59: Evaluate diagnostic accuracy Q(Ŷ, Y) across all modalities

60: Assess uncertainty calibration and clinical reliability

61: Check convergence criteria ||Ŷ(t+1) - Ŷ(t)|| < ε

62: t ← t + 1

63: until convergence or t > T_max

64:

65: // Generate comprehensive clinical outputs

66: Compute final diagnostic predictions Ŷ with confidence intervals

67: Select optimal feature combinations F* for each diagnostic task

68: Generate treatment outcome predictions $T_{\mathrm{outcomes}}$

69: Provide personalized risk stratification $R_{risk}$

70: return Ŷ, F*, $T_{\mathrm{outcomes}}, R_{\mathrm{risk}}$

**Algorithm 2:** Bio-Inspired Neutrosophic-Enzyme Framework

Input: Multi-modal data D = {X_clinical, X_radio, X_genetic}

Output: Diagnostic classification with uncertainty bounds

1: Initialize neutrosophic parameters for patient age group

2: FOR each region of interest r_i DO

3: Compute T(r_i), I(r_i), F(r_i) using Equations (1-3)

4: Apply spatial diffusion via Equation (4)

5: END FOR

6: Extract enzymatic features:

7: F_amylase ← Apply Equations (5-6) for caries detection

8: F_lysozyme ← Apply Equation (7) for infection patterns

9: Predict healing trajectory H(t) using Equations (8-10)

10: Optimize parameters θ* via genetic-immunological algorithm

11: Integrate evidence: E ← weighted_fusion(T, I, F, F_enzyme, H(t))

12: Generate diagnostic decision D̂ with confidence interval

13: RETURN {D̂, confidence_bounds, clinical_recommendations}

**Algorithm 3:** Enzyme-Inspired Feature Extraction

Input: Image regions I, patient demographics P, genetic data G

Output: Multi-enzyme feature vector F_integrated

1: Load age-specific enzymatic parameters from P

2: Initialize substrate concentrations from image intensities

3: FOR each enzymatic component DO

4: IF component = "amylase" THEN

5: Compute caries features via Michaelis-Menten kinetics (Eq. 5)

6: Apply age-dependent binding affinity (Eq. 6)

7: ELSE IF component = "lysozyme" THEN

8: Extract infection patterns via template matching (Eq. 7)

9: Adjust for immune system maturation based on age

10: END IF

11: END FOR

12: Calculate confidence weights from enzymatic activities

13: Fuse multi-enzyme features: F_integrated ← Σw_i·F_i/Σw_i

14: Compute cross-validation scores between enzyme responses

15: RETURN F_integrated with uncertainty quantification

**Algorithm 4** Multi-Objective Optimization with Immune Selection

Input: Feature space X, objectives O = {accuracy, uncertainty, clinical}

Output: Pareto-optimal solutions P*

1: Initialize population: P ← genetic(70%) + immune(30%) individuals

2: WHILE not converged DO

3: Evaluate fitness for all individuals using Equation (11)

4: Update Pareto front from non-dominated solutions

5: Apply negative selection via Equation (12)

6: Perform genetic operations: crossover, mutation

7: Execute immune operations: clonal selection, hypermutation

8: Implement territorial resource allocation

9: Replace population with elitism preservation

10: Check convergence: hypervolume improvement < 0.001

11: END WHILE

12: Select clinical solution from Pareto front

13: Validate on independent test set

14: RETURN optimized parameters and performance metrics
